# Sulfoglycolysis sustains *Eubacterium rectale* in low-fiber diets

**DOI:** 10.1016/j.jbc.2025.108320

**Published:** 2025-02-14

**Authors:** Mahima Sharma, Nicholas Pudlo, Michael A. Järvå, Arashdeep Kaur, Alan John, Laura Burchill, James P. Lingford, Ruwan Epa, Palika Abayakoon, Nichollas E. Scott, Johan P. Turkenburg, Gideon J. Davies, Eric C. Martens, Ethan D. Goddard-Borger, Spencer J. Williams

**Affiliations:** 1 York Structural Biology Laboratory, Department of Chemistry, University of York, York, United Kingdom; 2 Department of Microbiology and Immunology, University of Michigan Medical School, Ann Arbor, Michigan, USA; 3 ACRF Chemical Biology Division, The Walter and Eliza Hall Institute of Medical Research, Parkville, Victoria, Australia; 4 Department of Medical Biology, University of Melbourne, Parkville, Victoria, Australia; 5 School of Chemistry and Bio21 Molecular Science and Biotechnology Institute and University of Melbourne, Parkville, Victoria, Australia; 6 Department of Microbiology and Immunology, University of Melbourne at the Peter Doherty Institute for Infection and Immunity, Parkville, Victoria, Australia

**Keywords:** microbiome, prebiotics, glycosidase, carbohydrate metabolism, structural biology, bioinformatics

## Abstract

The production of short-chain fatty acids (SCFAs) by *Firmicutes* (*Bacillota*) within the human gastrointestinal tract is recognized as critical for gut health and the progression of a range of disease states. Firmicutes are the most diverse phylum of human gut bacteria, are highly studied, and are often specialized to degrade just a few polysaccharide substrates. Members of the Firmicutes include key bacteria that produce butyrate, an SCFA that is generally not produced by members of the other major phyla. Recently, it was shown that *Eubacterium rectale*, a widespread member of the *Firmicutes* belonging to the *Clostridiales* cluster XIVa, can grow on the unusual but ubiquitous plant-derived sugar SQ using a sulfoglycolytic sulfofructose transaldolase pathway. Here, we show that in addition to SQ, *E*. *rectale* can also grow on the SQ glycoside sulfoquinovosyl glycerol (SQGro). The 3D structure of the *E*. *rectale* sulfoquinovosidase (SftG) shares strong structural conservation with other carbohydrate-active enzyme family GH31 SQases. Using sequence-similarity networks, we provide new biological context to a conserved domain of unknown function protein SftX belonging to DUF4867, which is conserved in the sulfoglycolytic sulfofructose transaldolase pathway, and determine its 3D structure. Finally, with the aid of a synthetic mini-human microbiome reconstituted in germ-free mice, we show that an SQ dietary supplement can rescue *E*. *rectale* from population crashes that occur upon switching from a high-fiber to a low-fiber, high-fat diet. This suggests that SQ or SQGro has the potential as a prebiotic for promoting the maintenance of this important butyrate-producing bacterium within the colonic microbiota.

A complex community of bacteria inhabits the distal gut (1). This microbial community forages on diverse and complex carbohydrate substrates that are inert to host digestive enzymes within the jejunum and ileum. Bacteria of the colon disassemble and ferment these recalcitrant carbohydrates, which include polysaccharides and glycoconjugates, into short-chain fatty acids (SCFAs) that are readily absorbed by the host (2, 3). Bacterial SCFAs, such as butyrate, confer significant health benefits: they are a major source of energy for colonocytes (4), promote tight junction formation (5, 6), and drive the proliferation of regulatory T cells that keep autoimmune responses in check (7). A decrease in the number of SCFA-producing bacteria in the colon is correlated with the progression of diseases including ulcerative colitis (UC) (8, 9), Crohn’s disease (8, 10, 11), and cancer (12). We are only now beginning to discern whether changes in microbiota composition associated with the disease are a consequence of the disease or contribute to its underlying etiology. Testing hypotheses concerning the role of SFCA-producing microbial communities in disease etiology has been challenging, owing to a lack of tools to selectively manipulate the abundance of SCFA-producing bacteria in the colon.

*Eubacterium rectale* (*Agathobacter rectalis*) is a member of the *Firmicutes* (*Bacillota*) phylum of Gram-positive bacteria (13). *E*. *rectale* and other *Firmicutes* belonging to *Clostridiales* clusters IV and XIVa are the principal butyrate producers in the colon (3), and comprise a substantial part (10–40%) of the total bacteria in the gut (14). Among SCFAs, butyrate has received significant attention owing to its roles as a significant carbon (energy) source for colonocytes, its ability to modulate immune and inflammatory responses, and as a ligand for several G protein-coupled receptors (15). *Clostridiales*, including *E*. *rectale*, possess fewer complex carbohydrate degrading pathways than other generalists such as members of the phylum *Bacteroidota* and family Bifidobacteriaceae (16). Cross-feeding interactions have been identified that involve the utilization by *Clostridiales* members of either metabolic end-products or energy-rich fermentation products derived from complex carbohydrates that are produced by *Bacteroidota* bacteria (3). A dietary supplement that could directly sustain butyrate-producing bacteria, such as *E*. *rectale*, in the colon might assist in tackling questions surrounding the role of the microbiota in the aforementioned diseases and could have the potential to redress colonic dysbiosis and supporting fecal microbial transplant procedures that aspire to restore levels of butyrate-producing bacteria (17).

Recently, it was shown that *E*. *rectale* can degrade the sulfosugar sulfoquinovose (SQ) (18). SQ is a naturally occurring sulfosugar that is found as the head group of the sulfolipid sulfoquinovosyl diacylglyceride (SQDG), which itself is ubiquitous in photosynthetic tissues of plants, algae, dinoflagellates, and cyanobacteria (19). Incubation of fecal slurries from vegetarians with SQ led to an increase in levels of *E*. *rectale*, with the production of 2,3-dihydroxypropanesulfonate (DHPS) (20). DHPS in turn supported the growth of *Bilophila wadsworthia*, which metabolizes DHPS to form H_2_S (20). Thus, this pair of bacteria are collectively sustained by SQ and are sufficient to effect complete utilization of all carbon in this molecule within the human gut.

The catabolism of SQ by *E*. *rectale* is enabled by a gene cluster encoding a sulfoglycolytic sulfofructose transaldolase (sulfo-SFT) pathway (Fig. 1*A*) (18). The pathway involves an SQ isomerase (SftI) that converts SQ to 6-deoxy-6-sulfofructose (SF) and a transaldolase (SftT) (21) to catalyze metathesis of SF with glyceraldehyde-3-phosphate to form sulfolactaldehyde (SLA) and fructose-6-phosphate (F6P) (Fig. 1*B*). F6P enters glycolysis to support primary metabolism, while the SLA by-product is reduced to DHPS by an NADH-dependent reductase (SftR) and is exported by SftE. The sulfo-SFT gene cluster also contains a gene encoding a protein of the domain of unknown function (DUF) family 4867 termed SftX, whose abundance increases upon growth on SQ *versus* glucose, but for which the function is unknown (18).

**Figure 1:**
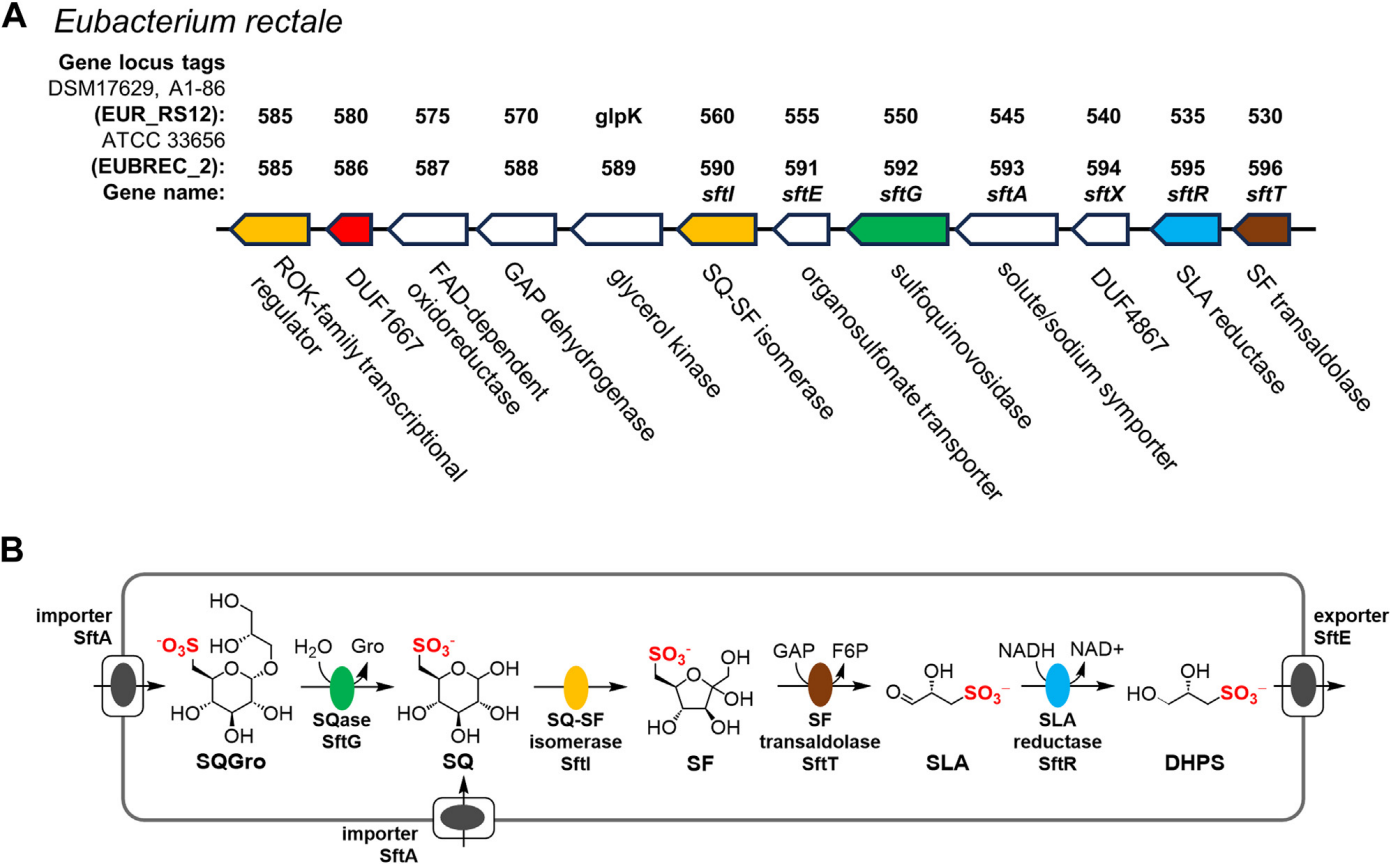
**Sulfoglycolytic sulfofructose transaldolase pathway for SQ utilization in *Eubacterium rectale* (*Agathobacter rectalis*)**. *A*, genetic loci of *E*. *rectale* strains DSM17629 and ATCC 33656 involved in sulfoglycolysis and the roles of their gene products. *B*, sulfoglycolytic sulfofructose transaldolase pathway in *E*. *rectale*.

*E*. *rectale* encodes within its sulfoglycolytic gene cluster a putative glycosidase termed a sulfoquinovosidase (SQase), SftG (22, 23). While *E*. *rectale* can grow on SQ in the laboratory, it is likely that in the gut niche, this bacterium utilizes this putative SQase to cleave SQDG, or more likely the deacylated form, sulfoquinovosyl glycerol (SQGro), which is produced by non-specific esterases in the mammalian digestive tract (24). The SQ released will then enter into the sulfo-SFT pathway. The sulfo-SFT gene cluster in *E*. *rectale* also encodes a glycerol kinase that may help in the utilization of glycerol released from hydrolysis of SQGro (18).

The existence of SQ utilization pathways in the SCFA-producing Firmicute *E*. *rectale* raises the prospect of using dietary modification with this specialized nutrient to manipulate their abundance in the gut. Here, we demonstrate using q-RT-PCR and comparative proteomics that growth of *E. rectale* on SQ causes an increase in mRNA expression and abundance of proteins in the sulfo-SFT pathway. We demonstrate that the *E*. *rectale* protein SftG encodes a functional SQase and determine its 3D structure, showing highly conserved residues that provide recognition of the sulfonate group of SQ. To gain insights into the role of the uncharacterized SftX DUF4867 protein in this pathway, we determine its 3D structure and reveal it to be a metalloprotein but with a function that remains enigmatic. By structural comparison and sequence-similarity network analysis of the DUF4867 family of proteins, we propose a possible role for these proteins as mutarotases/ring isomerases in ketose metabolism. Finally, we study the effect of SQ dietary supplements in a gnotobiotic mouse model in which animals were colonized with a human-like mini-microbiome comprised of 14 human gut bacteria, including *E*. *rectale*. We show that SQ supplementation supports high *E*. *rectale* populations upon switch from a high-fiber to a low-fiber diet, which otherwise leads to a dramatic decline in *E*. *rectale* population, that is, SQ functions as a targeted prebiotic for *E*. *rectale*.

## Results

### *E*. *rectale* utilizes SQ as sole carbon source

**Figure 2:**
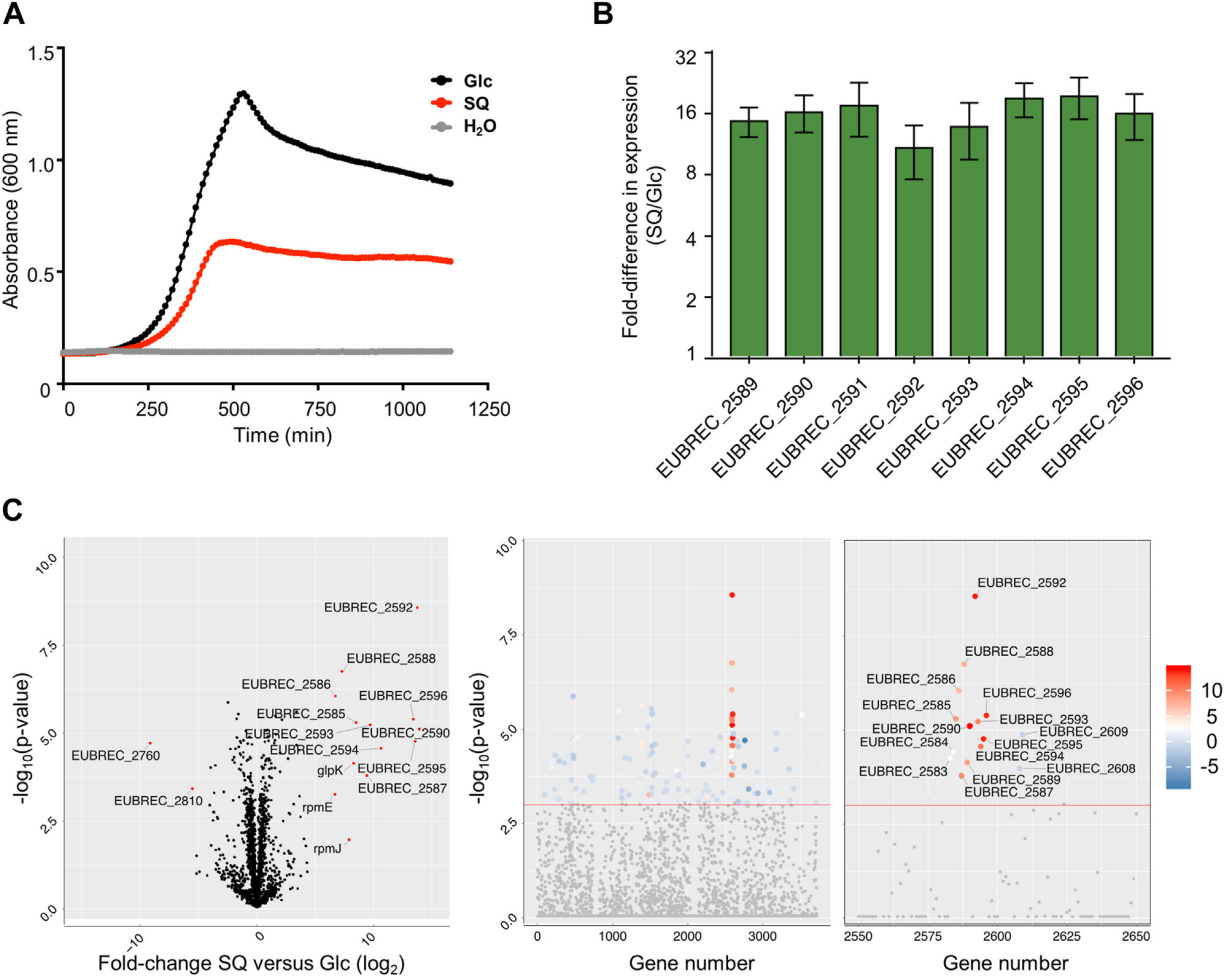
**SQ utilization by *Eubacterium rectale* (*Agathobacter rectalis*) strain ATCC 33656**. *A*, Absorbance at 600 nm of *E*. *rectale* cultures in minimal media supplemented with 5 mg mL^-1^ Glc or SQ. *B*, RT-q-PCR data for key genes in the putative SQ-utilization cluster. Error bars are standard deviation of the mean. *C*, Volcano and Manhattan plots of comparative proteomics data for the Glc vs SQ conditions.

*E*. *rectale* ATCC 33656 possesses a syntenic sulfo-SFT gene cluster to strain DSM 17629, A1-86 (Fig. 1*A*). Strain ATCC 33656 was grown in semi-defined media, which supports poor growth in the absence of a usable carbon source, supplemented with 5 mg mL^-1^ D-glucose (Glc) or SQ as the sole carbon source, and was monitored spectrophotometrically (Fig. 2*A*). The bacterium grew well on both Glc and SQ and at stationary phase, the optical density of the SQ culture was approximately half that of Glc culture, presumably because sulfoglycolysis of SQ yields only one C3 metabolite for primary metabolism, whereas Glc yields two (18).

Comparative proteomics on mid-log phase *E*. *rectale* cell pellets grown on SQ or Glc provided insights into changes in protein abundance occurring upon growth on SQ (Fig. 2*C*). These data identified gene products from a gene cluster comprised of EUBREC_2585 to 2596 as potentially responsible for SQ metabolism: these proteins experienced up to 1000-fold increase in abundance. This result was recapitulated at the transcript level: 16-fold increases in the transcripts for EUBREC_2589 to 2596 were observed by quantitative reverse transcription polymerase chain reaction (qRT-PCR), showing that changes in protein abundance likely occurred because of increased gene expression (Fig. 2*B*).

### EUBREC_2592 encodes a sulfoquinovosidase

**Figure 3:**
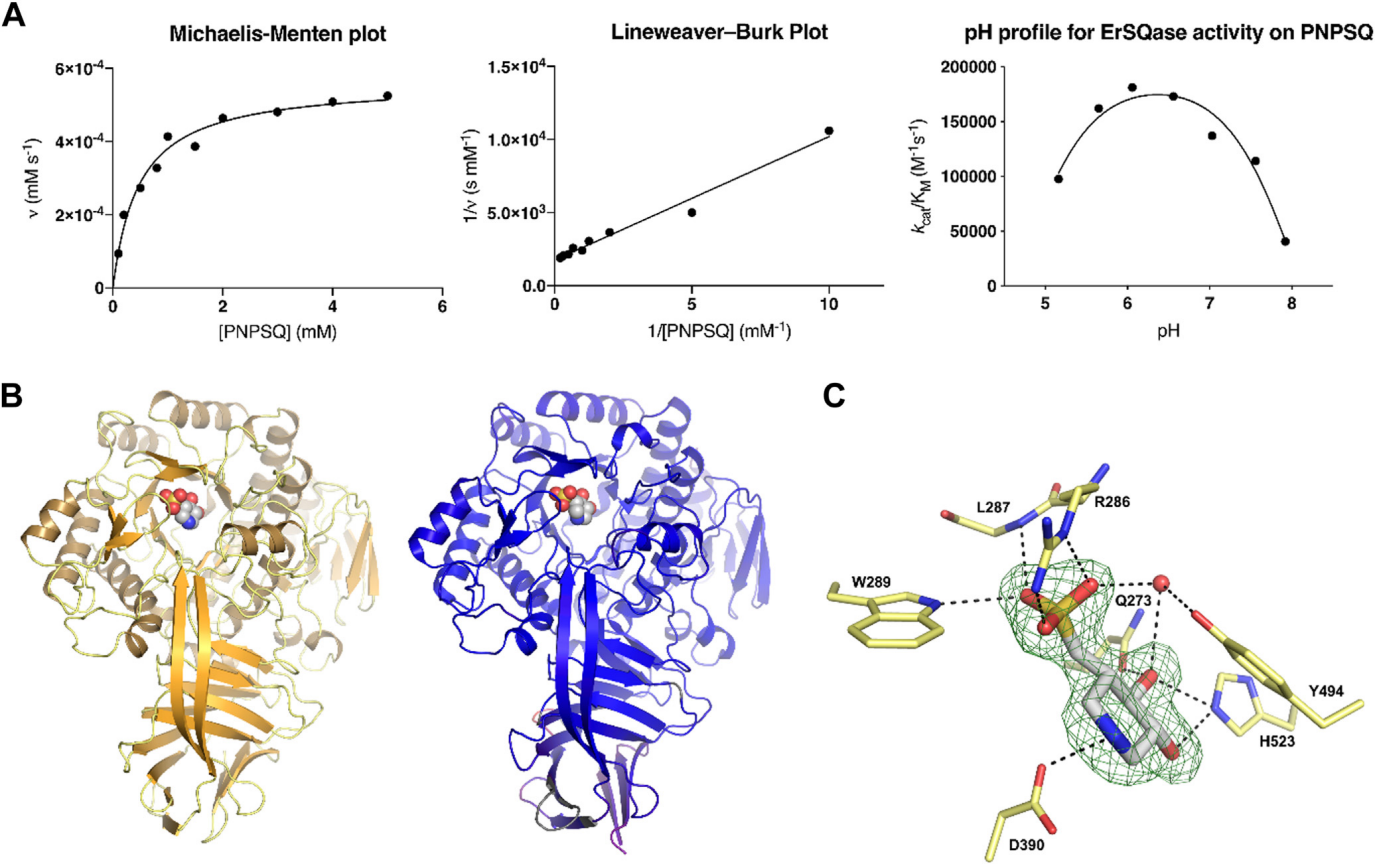
**Kinetic and structural analysis of *E*. *rectale* SftG SQase encoded by EUBREC_2592**. *A*, Michaelis-Menten and Lineweaver-Burk plots for SftG activity on pNP-SQ, as well as the pH dependence of activity. *B*, 3D structures of *E*. *rectale* SftG and *E*. *coli* YihQ (pdb 5OHT) SQases. *C*, the active site of SftG with bound IFGSQ and Fo-Fc contoured at 3σ.

To provide experimental validation of the putative SQase in the *E*. *rectale* sulfoglycolysis gene cluster, EUBREC_2592 encoding SftG was expressed, and the protein was purified from *Escherichia coli*. SftG cleaved the chromogenic SQase substrate 4-nitrophenyl sulfoquinovoside (pNPSQ), enabling assessment of the pH dependence of the enzyme and measurement of Michaelis-Menten kinetics (Fig. 3*A*). The kinetic properties of SftG are similar to those of the SQases from *E*. *coli* and *Agrobacterium tumefaciens* (Table 1) (22, 23). These latter enzymes, and likely SftG too, are retaining glycosidases that belong to the Carbohydrate Active Enzyme family GH31 and operating through a classical Koshland double-displacement mechanism involving a catalytic acid/base and nucleophile residue (Fig. S1) (22, 23).

**Table 1:** Comparison of *K*_M_, *k*_cat_, *k*_cat_/*K*_M_ values for three SQase enzymes

Enzyme	pH	*k*_cat_ (s^-1^)^a^	*K*_M_ (mM)^a^	*k*_cat_/*K*_M_ (M^-1^ s^-1^)^a^
*E*. *coli* YihQ (23)	7.25	14.3 ± 0.4	0.2215 ± 0.03	(6.4 ± 1.0) × 10^5^
*A*. *tumefaciens* SQase (22)	7.25	22.3 ± 0.6	0.21 ± 0.032	(1.1 ± 0.1) × 10^5^
*E*. *rectale* SftG	6.5	78.8 ± 3.9	0.478 ± 0.03	(1.65 ± 0.18) × 10^5^

a Errors are standard error mean.

SftG was co-crystallized with isofagomine-sulfoquinovose (IFGSQ) (22), an active-site-binding azasugar that inhibits SQases. The crystals obtained for this complex diffracted X-rays to a resolution of 1.9 Å (Fig. 3*B*, Table S2). The structural model built using these data revealed that the fold of SftG was highly similar to that of other structurally characterized SQases (Fig. 2*B*). The IFGSQ complexes of *E*. *rectale* SftG (PDB: 6PNR) and *E*. *coli* YihQ (PDB: 5OHT) have a Cα RMSD of 0.74 Å across 606 residues (1.69 Å across 661 residues, cutoff 2.0 Å), and with *A*. *tumefaciens* SQase (PDB: 5OHY) the Cα RMSD is 0.65 Å across 595 residues (1.77 Å across 655 residues, cutoff of 2.0 Å), as calculated using ‘A’ chains from each complex. In line with the putative transition state mimicry of IFGSQ, this ligand made similar interactions with SftG as were seen within complexes with *E*. *coli* YihQ and *A*. *tumefaciens* SQase (Figs. 3*C* and S1). A noteworthy difference between these three complexes is the conformation observed for the IFGSQ piperidine ring. In both *E*. *rectale* SftG and *A*. *tumefaciens* SQase, the IFGSQ adopts a conventional ^4^*C*_1_ chair conformation with the piperidine nitrogen hydrogen bonding to solvent (water) or cryopreservant (ethylene glycol), while in *E*. *coli* YihQ IFGSQ adopts a ^1^*S*_3_ skew conformation with the nitrogen interacting with the catalytic Asp405 residue in a manner that better mimicked the covalent intermediate. This reveals how subtle changes in active site solvation can influence ligand conformation on enzyme.

### A possible role for the DUF4867 protein family in ketose metabolism

SftX (encoded by EUBREC_2594) belongs to the DUF4867 (InterPro IPR032358) protein family. The role of this protein in the *E*. *rectale* sulfo-SFT pathway is unclear. The main chemical steps in the pathway have enzymes already assigned, and no proteins within the DUF4867 family have an assigned function. Among the closest characterized sequence-related proteins are the ureidoglycolate lyases (IPR007247), typified by *E*. *coli* AllA (25). Ureidoglycolate lyases are Ni^2+^-binding metalloenzymes that catalyze the elimination of urea and glycolate from ureidoglycolate (26). We used the Genome Neighborhood Tool available through the Enzyme Function Initiative to explore the loci adjacent to each DUF4867-encoding gene to infer the possible biological context of each DUF4867 sequence (27). The DUF4867 members were downloaded from the InterPro database and used to create a series of sequence similarity networks (SSNs) using an all-by-all pairwise BLAST with varying alignment scores corresponding to different sequence alignment score thresholds (Fig. S2). In these SSNs, each node is a DUF4867 member, and a line connects nodes if the sequence alignment is greater than the alignment score threshold. The most promising three SSNs were each used to generate genome neighborhood networks (GNNs), which contain hub-spoke clusters showing common neighborhood gene-encoding PFAMs (Fig. S3). The SSN with AS = 70 was chosen because the resultant GNNs gave a meaningful set of possible biochemical pathways.

**Figure 4:**
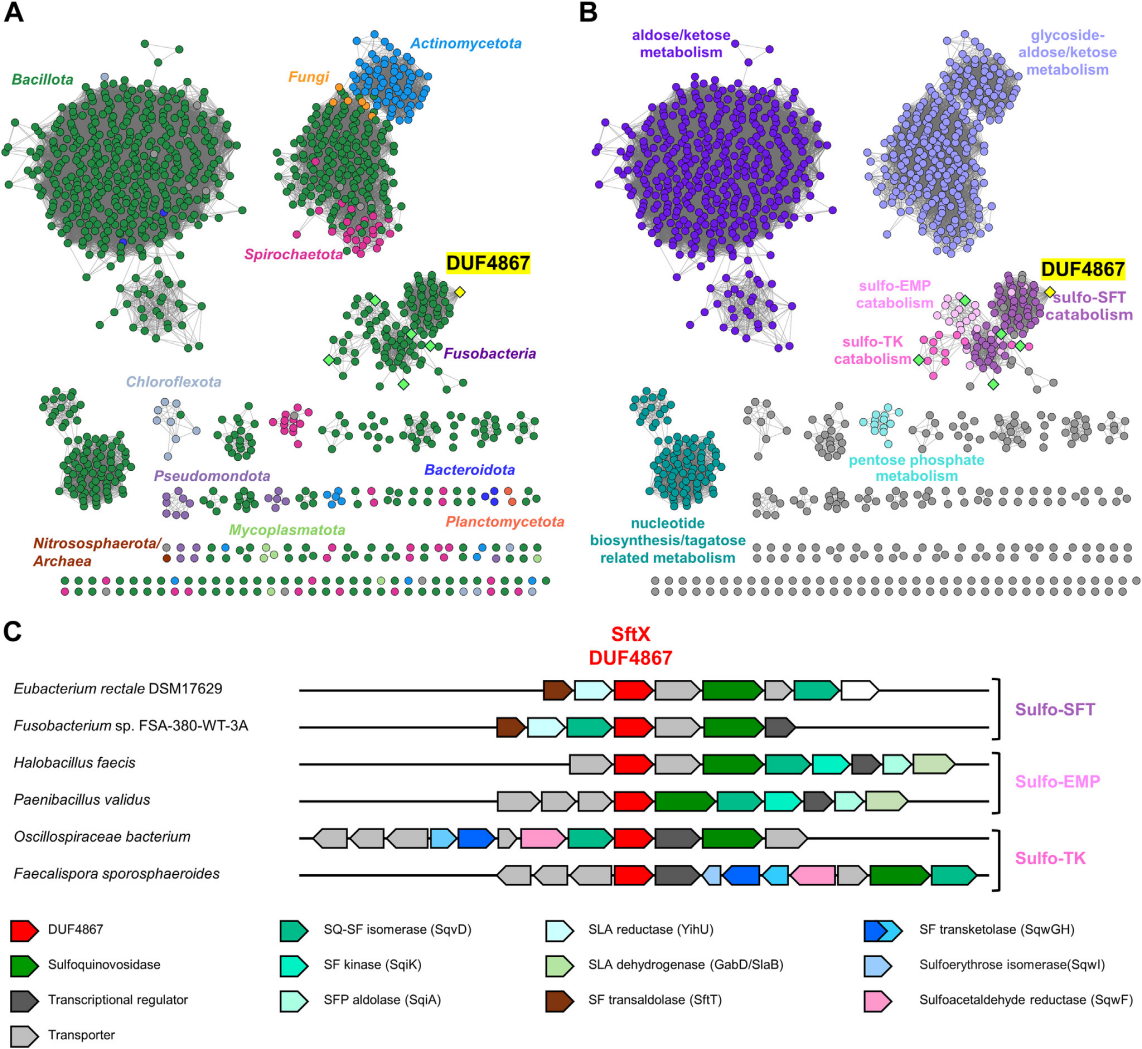
**Sequence similarity network (SSN) analysis of DUF4867 family proteins, at alignment score 75**. *A*, SSN was colored based on the taxonomy of organisms harboring the gene encoding the DUF4867 protein. *B*, SSN was colored based on the genetic context of the DUF4867 gene within proposed sulfoglycolytic (sulfo-EMP, sulfo-SFT, and sulfo-TK) pathways, or aldose/ketose, nucleotide biosynthesis/tagatose-related, pentose phosphate, glycoside-aldose/ketose metabolism. SSNs were visualized with Cytoscape (62). The *yellow* diamond node is DUF4867 from *Eubacterium rectale*. Lime *green* diamond nodes are for organisms from part C. *C*, genome neighborhood diagrams for DUF members with sulfoglycolytic degrading genes within ± 10 open reading frame window. Protein accession codes for DUF4867 proteins: *Fusobacterium* sp. FSA-380-WT-3A (A0A848C2V3), *Halobacillus faecis* (A0A511WTK5), *Paenibacillus validus* (A0A7X2ZGL5), Oscillospiraceae *bacterium* (A0A356AKR7), *Faecalispora sporosphaeroides* (A0A928Q3Q3).

*E*. *rectale* DUF4867 member SftX is found in a medium-sized cluster (*n* = 127 sequences) (Fig. 4, *A* and *B*). The genome neighborhood of the members of this cluster encodes proteins of sulfoglycolytic pathways including sulfo-SFT (*n* = 86/127; PFAM codes: PF00923, PF02952, PF03446, and PF14833), sulfo-TK (*n* = 15/127; PF02502, PF02779, PF02780, PF00456, and PF00465), and sulfo-EMP (*n* = 23/127; PF02952, PF00365, PF01116, PF02826, PF03446, and PF14833; these correspond to sulfo-EMP2 PFAMs (28)) pathways. Some example genome neighborhood diagrams of bacteria containing sulfo-SFT, sulfo-EMP, and sulfo-TK gene clusters are shown in Figure 4*C*.

The largest cluster in the DUF4867 SSN was comprised of 557 sequences (Fig. 4, *A* and *B*). Most sequences (434/557) are found in genome neighborhoods encoding pathways assigned to aldose/ketose metabolism, including PF02952 (fucose isomerase family), and PF00370-PF02782 (FGGY carbohydrate kinase family). The second largest cluster was comprised of 298 sequences mainly found in genome neighborhoods encoding pathways assigned as glycoside-aldose/ketose metabolism (61/298), including PF00232 (glycoside hydrolase family 1), PF02952 (fucose isomerase family), and PF00370-PF02782 (FGGY carbohydrate kinase family). A smaller cluster was comprised of 117 sequences mainly found in genome neighborhoods encoding pathways assigned as nucleotide biosynthesis/tagatose-related metabolism, including PF01263 (*n* = 61/117; aldose epimerase family), PF01087-PF02744 (*n* = 34/117; galactose-1-phosphate uridyl transferase family), and PF16363 (*n* = 34/117; GDP-mannose 4,6 dehydratase family). A small cluster of 14 sequences was mainly found in genome neighborhoods encoding pathways assigned as pentose phosphate pathway (*n* = 8/14), including PF02502 (ribose/galactose isomerase family), PF02779-PF02780 (transketolase family), PF00456 (transketolase, thiamine diphosphate binding domain) and PF00465 (iron-containing alcohol dehydrogenase family). While the majority of DUF4867 members are single-domain proteins, 10 DUF4867 members of InterPro IPR032358 are part of multidomain proteins assembled as IPR018484–IPR018485–IPR032358, where the first two domains are N- and C-terminal domains of ATP-dependent FGGY carbohydrate kinases that act on a range of alditols and ketoses.

### 3D structure of a DUF4867 protein family member from *Bacillus megaterium*

Efforts to crystallize recombinant *E*. *rectale* SftX were unsuccessful, so we turned to examining homologs to elucidate the representative 3D structure of the DUF4867 family. *Bacillus megaterium* encodes a functional sulfo-SFT pathway and the corresponding gene cluster contains a DUF4867 family member, SqvB, with 51% sequence identity with *E*. *rectale* SftX (28). *B*. *megaterium* SqvB was cloned, expressed in *E*. *coli*, purified, and crystallized. The structure was initially solved using 1.5 Å resolution diffraction data in tandem with 2.3 Å selenomethionine data, the latter collected at a wavelength to optimize the *f*″ signal from the selenium anomalous scatterers. Native and SeMet-labeled SqvB crystallized in the P3_2_21 space group with two molecules in the asymmetric unit (Table S3). PISA analysis shows that the two SqvB bury 3353 Å^2^ at the dimer interface, accounting for 16% of the total surface area of the assembly. Determination of the molecular weight of the predominant protein species in solution using size exclusion chromatography-multi angle laser light scattering (SEC-MALLS) revealed that SqvB exists as a homodimer in solution (Fig. S4).

**Figure 5:**
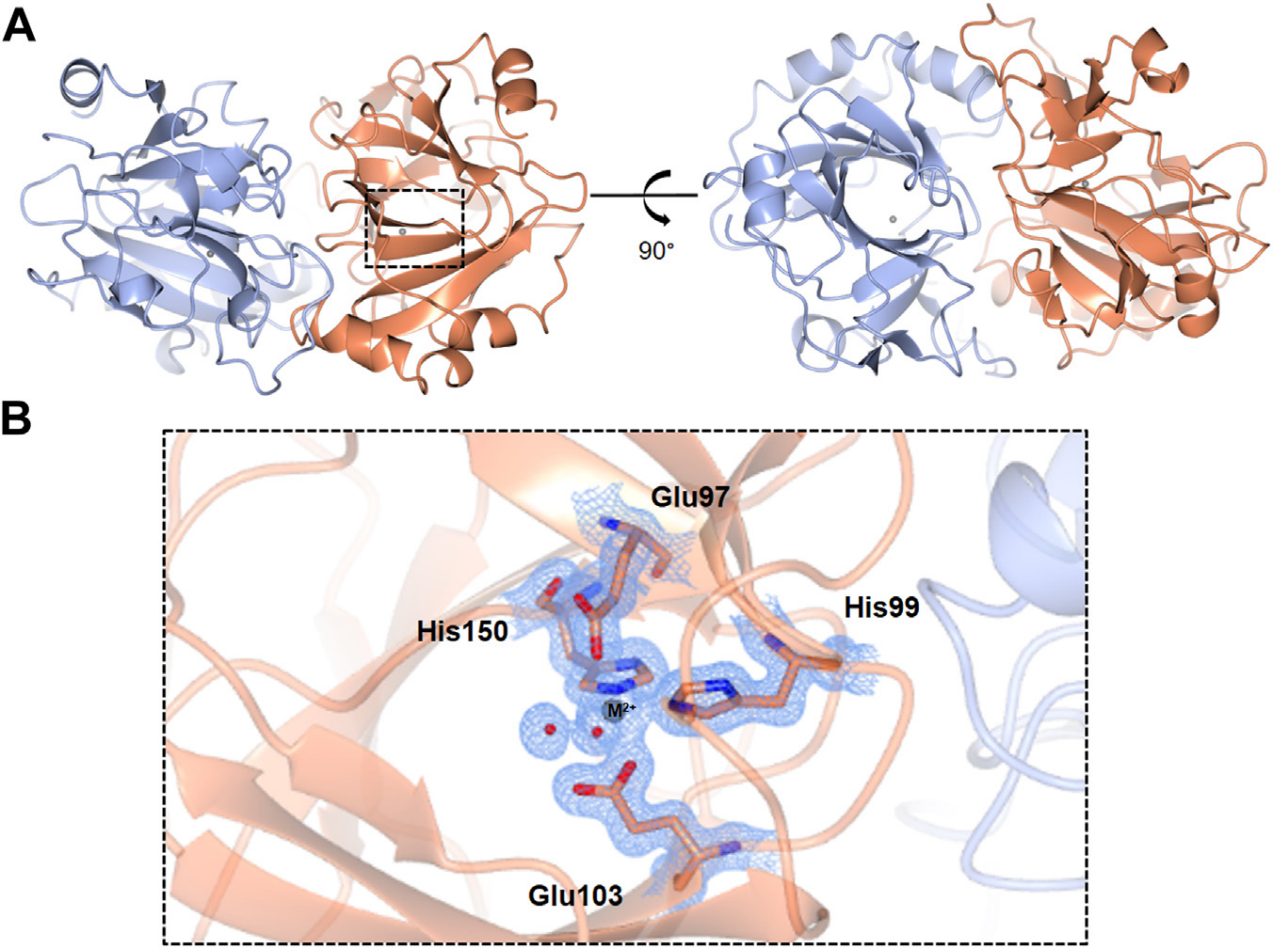
**3D structures of a DUF4867 family member, SqvD, from the sulfo-SFT gene of *Bacillus megaterium***. *A*, quaternary structure of metal-bound SqvD, with monomers A and B showed in coral and ice *blue* in ribbon format. *B*, close-up view of the metal binding site, modeled with Fe, showing two bound water molecules (red). Electron density corresponds to 2Fo-Fc map (in *blue*) at levels of 1σ.

*B*. *megaterium* SqvB adopts an α/β fold with a central, characteristic β-barrel and belongs to the cupin superfamily (cupin from latin *cupa*, a barrel) (Fig. 5*A*). X-ray fluorescence (XRF) scanning using a beam energy of 11 to 13 keV confirmed the presence of Fe (*Ekα* ∼7 keV in the emission spectrum) and Zn (*Ekα* ∼8.6 keV in the emission spectrum). These results were corroborated by quantitative inductively coupled plasma-optical emission spectroscopy analysis of BmDUF4867, which detected Fe and Zn in a solution of the protein (Table S4). Based on the XRF mapping and X-ray diffraction datasets analyzed for the anomalous signal above and below the Fe absorption edge (Fig. S5), the metal was modeled as Fe at an occupancy of 1 at the center of the β-barrel in both subunits. The metal ion is buried at the core of the β-barrel and binds in an octahedral geometry to 2His-2Glu motif: His150 (Nε2 at 2.1 Å), His99 (Nε2 at 2.1 Å), Glu103 (Oε at 2.0 Å), Glu97 (Oε 2.3 Å) and two water molecules present distances of 2.0 and 2.4 Å (Figs. 5*B*, S6). Pairwise DALI analysis of the closest structural homologs of DUF4867 identifies a putative ureidoglycolate lyase from *P*. *putida* (PDB ID: 2BDR with a DALI Z score of 11.2 and 2.8 Å root mean square deviation (rmsd) and 19% sequence identity), a putative cysteine dioxygenase from *B*. *subtilis* (PDB ID: 4QM8 with a DALI Z score of 8.5 and 2.7 Å rmsd and only 10% sequence ID), and a 3-hydroxyanthrilate-3,4-dioxygenase from *Cupriavidus metallidurans* (PDB ID: 6BVQ, DALI Z score of 7.3, 3.4 Å rmsd and 12% sequence ID).

### Investigation of possible enzymatic functions for DUF4867 family members

Members of the cupin superfamily adopt a variety of oligomeric states and fulfill functions and activities ranging from epimerases to lyases, as well as transcription factors and storage proteins in plant seeds (Fig. S7) (29). We speculated that the *E*. *rectale* or *B*. *megaterium* DUF4867 could play a role in anomerization of SQ or SF. Therefore, we soaked a crystal of SqvB with SF and could detect clear electron density for a ligand at the center of the cupin barrel. The metal (Fe) was modeled at the active center bound to the same amino acid residues as before, namely His150 (Nε2 at 2.0 Å), His99 (Nε2 at 2.1 Å), Glu103 at 2.0 Å, and Glu97 (1.9 Å). We observed that the water molecules bound at the metal center previously observed in the native structure were clearly displaced, the omit map (generated after modeling in the metal and solute) showing the additional, clear density of a ligand bound to the metal. However, we were unable to unambiguously model either SF or HEPES buffer (used in the crystal growth conditions) and the structure was deposited without modeling a sulfonate ligand (Fig. S8).

The role of DUF4867 family members in the sulfo-SFT pathway has not been determined. Our SSN analysis shows that homologs of genes encoding DUF4867 family members are also found in gene clusters that appear to encode other sulfoglycolytic pathways including the sulfo-EMP and sulfo-TK pathways. In all cases, there is no obvious missing enzymatic activity, and the existence of sulfo-EMP and sulfo-TK pathways that lack a DUF4867 encoding gene suggests that its contribution may not be essential. Given that the proteins with the closest sequence similarity that have been experimentally characterized, namely AllA and the ureidoglycolate lyases catalyze elimination reactions, we speculated that the DUF4867 family of proteins may catalyze the isomerization of aldopyranoses or ketofuranoses between anomers and the acyclic keto-forms. We, therefore, examined whether SftX could catalyze the mutarotation of SQ or SF using an NMR assay based on chemical exchange spectroscopy (30). However, we could not detect any rate enhancement of mutarotation in the presence of SftX, although this assay was limited to non-paramagnetic metal ions. We therefore also examined whether SQ or SF could bind *Bm*SqvD using nanoDSF but did not observe any change in melting temperature with 10 mM of either ligand (Fig. S9). Another hypothesis is that SftX may act as a kinase, to produce SFP, which could be an alternative substrate for the transaldolase in the sulfo-SFT pathway. However, incubation of SF and ATP with SftX did not lead to formation of SFP.

### SQ dietary supplements can sustain *E*. *rectale* populations in a low-fiber diet

**Figure 6:**
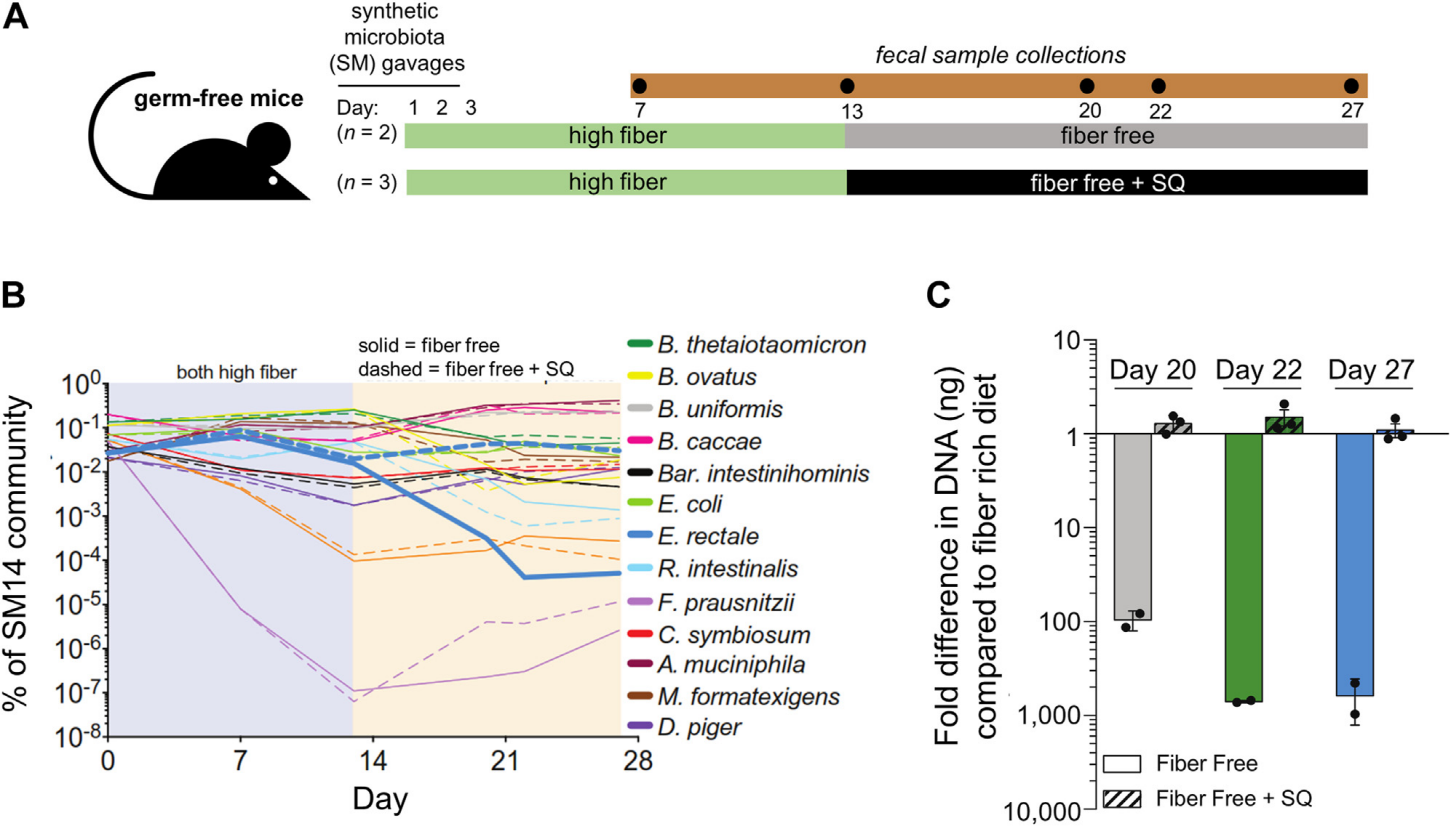
**Dietary SQ supplements rescue the *E*. *rectale* population in a low-fiber diet in female Swiss-Webster mice with a humanized microbiome**. *A*, experimental design. There were n = 2 mice in the fiber-free group, and n = 3 mice in the fiber-free + SQ group. *B*, changes in bacterial abundance in fecal samples with respect to time. *C*, fold difference of *E*. *rectale* in low-fiber diets with and without SQ supplementation. Error bars are the standard deviation of the mean and data points are the values detected within each mouse.

We next sought to determine if SQ can selectively enhance the *E*. *rectale* population *in vivo*. Gnotobiotic mice were inoculated with 14 bacterial species that broadly represent the taxonomic diversity and metabolic potential to degrade dietary fiber and host mucosal glycans present in a more complex human microbiota, as previously described (31). These animals were fed a high plant-fiber chow for 2 weeks to establish the 14-species synthetic microbiota, which was confirmed by qRT-PCR with species-specific primers on fecal samples from each animal (Fig. 6*A*). Mice were then switched to a low-fiber diet with or without SQ supplementation (1% w/v), provided *ad libitum* to the animals in their drinking water. Changes in the relative abundance of each bacterial strain were quantitated by qRT-PCR analysis of the fecal samples of each animal at days 0, 7, 13, 20, 22, and 27 (Fig. 6*B*). Of the 14 bacterial species present in the synthetic microbiota, the greatest difference between the two animal cohorts that was associated with SQ consumption was observed for *E*. *rectale*. In untreated animals, the abundance of butyrate-producing *E*. *rectale* dropped approx. 1000-fold in the first week of low fiber feeding and remained low until day 27, while the abundance in SQ-treated animals remained unchanged (Fig. 6*C*).

The effect of SQ was limited to *E*. *rectale*. SQ metabolic pathways have been reported in *Faecalibacterium prausnitzii* (sulfo-SFT) (20), *Roseburia intestinalis* (sulfo-SFT) (20), *Clostridium symbiosum* (sulfo-SFT) (20), and *E*. *coli* (sulfo-EMP) (32). However, the human-derived commensal *E*. *coli* HS (33), *F*. *prausnitzii* DSM 17677 A2-165 (34), and *R*. *intestinalis* L1-82 (35) strains used in our synthetic microbiota do not encode the full set of genes for a complete sulfoglycolytic pathways. On the other hand, *C*. *symbiosum* strain ATCC 14940 (36) does contain a complete set of genes encoding a sulfo-SFT pathway, and *Desulfovibrio piger* ATCC 29098 has been reported to contain a pathway enabling the use of DHPS for anaerobic sulfite respiration (20), but neither strain underwent a significant change in abundance in the diet supplemented with SQ.

## Discussion

The human gut harbors a diverse community of bacteria, predominantly from five major phyla: *Bacillota* (*Firmicutes*), *Verrucomicrobiota*, *Bacteroidota* (*Bacteroidetes*), *Actinomycetota* (*Actinobacteria*), and *Pseudomonadota* (*Proteobacteria*) (1). The distal gut, in particular, hosts the highest abundance of these bacteria, with large populations of obligate anaerobes from the *Bacteroidota* and *Bacillota* phyla, with the latter including *E*. *rectale*. In contrast, facultative anaerobes like *E*. *coli* are much less abundant (37). Collectively, the gut microbiota provides a range of nutritional and functional benefits to the host and is also implicated in various disease states. Many members of the gut microbiota produce short-chain fatty acids, including acetate, propionate, isobutyrate, and butyrate. Butyrate has been identified as a key mediator of generally beneficial processes, with *E*. *rectale* recognized as a significant contributor to its production (38, 39). The presence of *E*. *rectale* was indirectly required for exclusive enteral nutrition-dependent production of another beneficial branched short-chain fatty acid, isobutyrate (40).

Prior studies identified a sulfo-SFT pathway for SQ catabolism in *E*. *rectale* (18). We extended this work to demonstrate that *E*. *rectale* possesses a functional SQase that is expressed upon growth on SQ. This is significant because *E*. *rectale* does not utilize many dietary carbohydrates. Our analyses of the *E*. *rectale* ATCC 44656 genome revealed the presence of just 42 predicted GHs from 21 different GH families, as defined by the CAZy sequence-based classification system (www.cazy.org) (Table S4) (25). Consideration of the predicted biochemical function of these putative carbohydrate-active enzymes and their associated gene clusters led to the identification of several gene clusters likely to be involved suggesting that *E*. *rectale* can catabolize α-glucans, fructans, xylans, arabinoxylans, and cellobiose (26) in addition to SQ glycosides, such as SQDG or SQGro. Indeed, all these polysaccharides have been reported as substrates that can sustain the growth of *E*. *rectale* DSM 17629, A1-86 (18, 26). We also identified gene clusters that may support the catabolism of other carbohydrates that have not been explored as substrates for *E*. *rectale* DSM 17629, A1-86, including, sucrose, raffinose, and lacto-*N*-biose or other human milk oligosaccharides. The role of these dietary oligosaccharides in supporting E. *rectale* populations within the digestive tract remains to be explored.

The *E*. *rectale* sulfo-SFT gene cluster contains an uncharacterized open reading frame encoding a DUF4867 family protein. We determined the 3D structure of a homologous DUF4867 protein from *B*. *megaterium*, revealing it to belong to the cupin superfamily. Bioinformatics analysis showed that DUF4867 homologs are present in gene clusters encoding other sulfoglycolytic pathways, including the sulfo-EMP and sulfo-TK pathways. However, for all these pathways, enzymes have already been identified that catalyze all the key chemical steps, leaving the role of the DUF4867 proteins unclear. The only chemical species common to all three sulfoglycolytic pathways are SQ and SF, suggesting that if DUF4867 is an enzyme, it is likely to act on either of these compounds. Possible activities for DUF4867 proteins could include functioning as SQ or SF mutarotases to interconvert α- and β-anomers, and/or catalyzing the ring-opening reaction of sulfofructofuranose to the acyclic form, which is a substrate for SF aldolase or SF transketolase in the sulfo-SFT or sulfo-TK pathways. However, we were unable to detect activity upon SQ or SF using chemical exchange spectroscopy. Notably, the closest related protein with a known function is a Ni^2+^-dependent ureidoglycolate lyase (26). We were unable to test Ni^2+^ as a cofactor because it is diamagnetic and interferes with NMR experiments. This work bears comparison with the pectin-degrading protein KdgF (InterPro IPR025499), which is a metal-dependent enzyme with a cupin fold that catalyzes the conversion of 4,5-unsaturated uronates to acyclic 5-keto-4-deoxy-uronates (41, 42). The proposed mechanisms of ureidoglycolate lyase, KdgF, and the ring-opening reactions of SQ and SF all involve identical bond-making and breaking steps suggesting a possible conserved mechanism (Fig. S10).

To investigate the potential of SQ as a prebiotic targeting *E*. *rectale*, we used a previously developed model system (31) in which a synthetic microbiota, assembled from 14 fully sequenced human gut bacteria representing five bacterial phyla, was implanted into gnotobiotic mice. When fed a high-fiber diet (containing starch and fiber from corn, soybean, wheat, oat, and alfalfa), this microbiota remains stable and persists for extended periods. It was shown that switching from a high-fiber diet to a fiber-free diet results in a rapid increase in the abundance of mucin-degrading bacteria like *A*. *muciniphila* and *B*. *caccae*, while levels of polysaccharide-specializing communities like *B*. *ovatus* and *E*. *rectale* rapidly decline, with a return to the high-fiber diet allowing for their rapid recovery (31).

**Figure 7:**
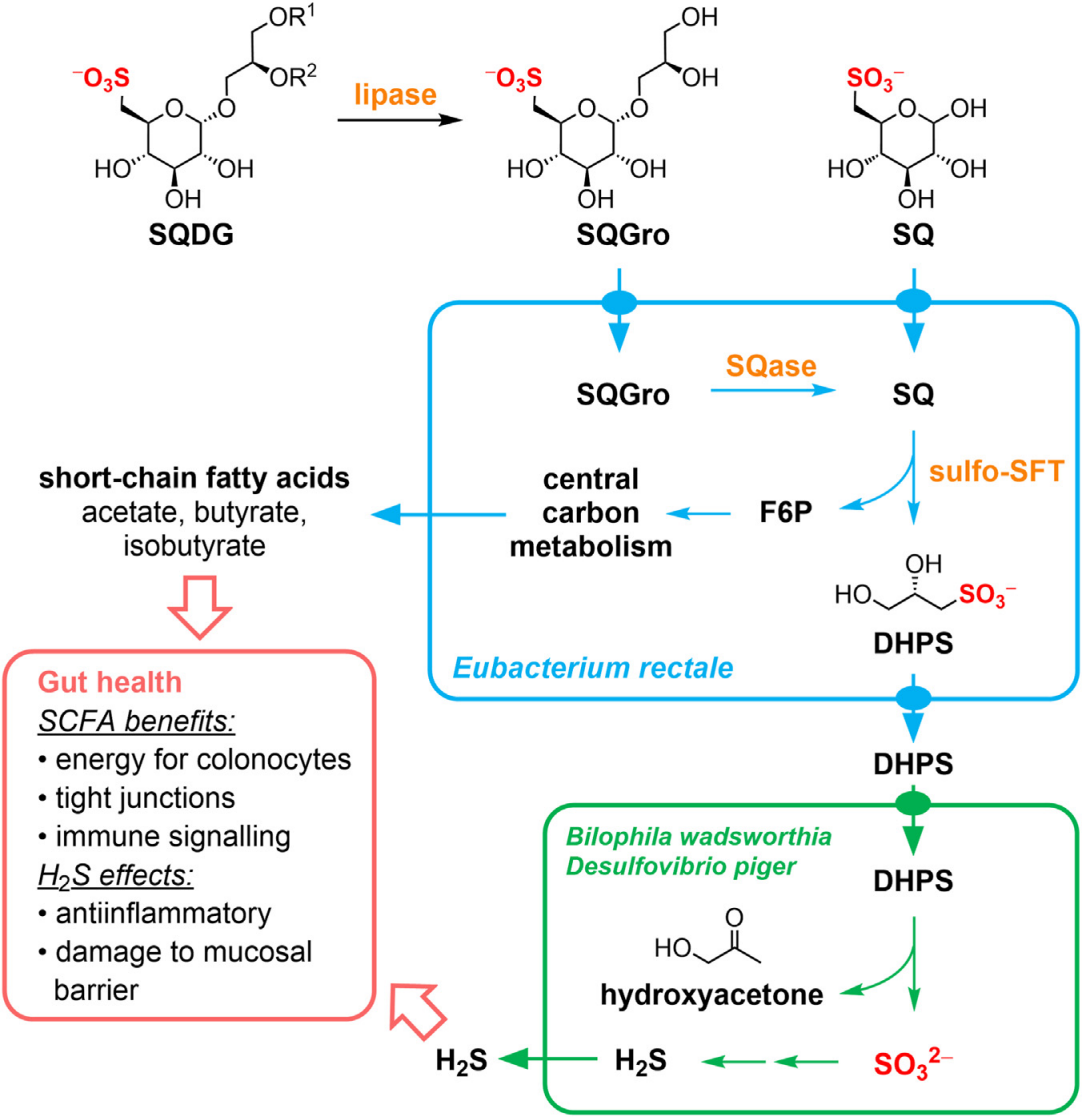
**Metabolism of SQ and SQ glycosides by sulfoglycolytic and sulfidogenic bacteria.** Sulfoquinovosyl diacylglycerol (SQDG) is delipidated by gut lipases to give sulfoquinovosylglycerol (SQGro). SQGro or SQ are imported and the former cleaved by SQase SftG. SQ breakdown through the sulfoglycolytic sulfofructose transaldolase pathway (sulfo-SFP) produces fructose-6-phosphate (F6P) that enters central carbon metabolism, resulting in the production of short-chain fatty acids, and DHPS, which is exported. DHPS-metabolising bacteria such as *Bilophila wadsworthia* and *Desulfovibrio piger* take up DHPS and by sulfolysis produce sulfite and hydrogen sulfide (H_2_S). SCFAs have beneficial effects on gut health, while H_2_S has multifaceted effects on the gut.

In our study, when mice harboring this 14-member synthetic microbiota were switched from a high-fiber to a low-fiber diet, we observed similar changes. However, when a separate group of mice was fed the low-fiber diet supplemented with SQ, the levels of *E*. *rectale* were selectively preserved. These data indicate that SQ has prebiotic capabilities that target *E*. *rectale* (Fig. 7). Interestingly, no effect was observed with *E*. *coli*. Previous studies have shown that *B*. *wadsworthia* can utilize DHPS released by sulfoglycolytic metabolism of SQ, forming hydrogen sulfide, which has been implicated in disparate effects on host health (20). Our 14-member microbiota lacks *B*. *wadsworthia*, but contains *D*. *piger*, which is proposed to be able to catabolize DHPS for anaerobic respiration (20), but no significant changes were seen in its abundance on the SQ-supplemented diet. Future work could expand the synthetic microbiota to include *B*. *wadsworthia* to study the effects of SQ supplementation on community population dynamics and gut health.

## Conclusion

This study corroborates previous findings that *E*. *rectale* can metabolize the plant sulfosugar sulfoquinovose (SQ) using the sulfoglycolytic sulfofructose transaldolase pathway. We characterized the 3D structure and function of the sulfoquinovosidase SftG, confirming its role in cleaving SQ glycosides, which are likely dietary sources of this sulfosugar. Furthermore, we determined the 3D structure of the DUF4867 protein SftX, revealing it to be a member of the cupin family. Although its function remains unclear, we observed its widespread distribution among various sulfoglycolytic pathways, suggesting a role in the isomerization of sulfofructose. We also show that dietary supplementation with SQ in gnotobiotic mice colonized with synthetic microbiota can effectively maintain *E*. *rectale* levels on a low-fiber diet. This highlights the potential of SQ as a targeted prebiotic to promote gut health by supporting butyrate-producing bacteria. In the longer term, possible sources of SQ for dietary supplementation could include SQDG-enriched extracts from algae or plants, which could be further processed to SQGro, or as used here, SQ obtained by chemical synthesis. Almost half of more than 2000 *E*. *rectale* genomes (from metagenome-assembled genomes) encode the sulfo-SFT pathway (20), suggesting that SQ could represent a useful tool for manipulating *E*. *rectale* levels in the gut. These results lay the groundwork for further investigations into dietary interventions aimed at modulating the gut microbiota and addressing colonic dysbiosis.

## Experimental procedures

### Materials

Sulfoquinovose (CAS registry number 3458–06–8) was obtained from MCAT GmbH. IFGSQ was synthesized as described (22) and PNPSQ was synthesized as described (23).

### Bacterial strains and culturing

Bacterial members of the 14-member synthetic microbiota (SM), including *E*. *rectale*, were grown in an anaerobic chamber (Coy Lab Products, Grass Lake, MI) at 37 °C under an atmosphere of 10% H_2_, 5% CO_2_, and 85% N_2_ as previously described (31). For *E*. *rectale* growth measurements on SQ, cells were grown in 5 ml YCFA medium containing 0.5% glucose overnight. A 1 ml aliquot was pelleted by centrifugation and the spent media was removed. Cells were resuspended in 1 ml of 2X YCFA containing no carbohydrate source and diluted 1:50 into 2X YCFA-no carbohydrate. A 100 μl aliquot of washed cells was added to a 96-well plate containing 100 μl of 2X carbohydrate stocks (1%), which were glucose or SQ (negative controls were added to water only). The resulting 1X cultures therefore contained a 1:100 final cell dilution and 0.5% final carbohydrate concentration and growth was monitored at 10 min intervals using a microplate stacking device and coupled absorbance reader (Biotek Instruments, Winooski, VT).

### RNA extraction and transcript measurements by qPCR

Total RNA was extracted from triplicate cell cultures grown either on glucose or SQ using a modified phenol-chloroform procedure. *E*. *rectale* was grown to mid-log phase in YCFA medium containing either 0.5% glucose or SQ. Cells were pelleted, and supernatants were removed and resuspended in 1 ml of RNA Protect Bacteria Reagent (Qiagen). After incubating for 5 min at room temperature (RT), cells were centrifuged at 7000 x g for 10 min at room temperature, supernatants were decanted, and the pellets were stored at −80 °C until RNA was extracted. Cell pellets were thawed on ice, resuspended in 200 μl TE (10 mM Tris, 1 mM EDTA, pH 8.0) buffer with lysozyme (1 mg mL^-1^) and incubated at room temperature for 2 min prior to adding 700 μl RLT Buffer (Qiagen) with 10 μl mL^-1^ β-mercaptoethanol (Sigma). After mixing thoroughly, 500 μl phenol:chloroform:isoamyl alcohol (125:24:1; pH 4.5) was added and the resulting mixtures were mixed by inversion. Following bead beating for 3 min, samples were centrifuged at 18,000 x g for 3 min at 4 °C. The aqueous phase was removed into a new RNase-free tube and another 500 μl phenol:chloroform: isoamyl alcohol was added, mixed, and centrifuged as above. The aqueous phase was removed to a new tube, 0.1 volume of 3 M sodium acetate (pH 5.5) and 600 μl cold 100% isopropanol were added and the resulting mixtures were mixed by inversion. RNA was precipitated at −80 °C for 20 min before centrifuging at 18,000 x g for 20 min at 4 °C. Supernatants were discarded and RNA pellets were washed with 70% isopropanol prior to centrifuging at 18,000 x g for 5 min at 4 °C. Supernatants were discarded and pellets were dried at room temperature before resuspending in 86 μl nuclease-free water. RNA was subjected to 4 μl DNase I treatment (NEB) for 1 h at 37 °C, followed by the addition of 4 μl 0.5 M EDTA to terminate DNase activity. RNA was re-precipitated and pelleted as above. Finally, RNA pellets were washed with 1.3 ml 70% isopropanol, prior to drying and resuspension in 50 μl RNase-free water. Total RNA was quantified using a Nanodrop (Thermo Fisher). cDNA was prepared from 1 μg of total RNA with SuperScript III reverse transcriptase (Invitrogen) and gene-specific primers used to quantify transcripts with a homemade qPCR mix (43). Primers that target the 16S rRNA transcript were used to normalized cycle threshold values for gene-specific transcripts and compare expression between YCFA + SQV and YCFA + glucose as a fold-change (SQV/glucose).

### Gnotobiotic mouse experiment

The mouse experiment was approved by the University Committee on Use and Care of Animals at the University of Michigan. Six-to-eight-week-old, germfree female Swiss-Webster mice (*n* = 5) were gavaged with the synthetic microbiota mixture containing 14 species and fed a normal chow (fiber-rich) lab diet (LabDiet 5010, LabDiet, St Louis, MO) for 2 weeks. Two mice were switched to a fiber-free diet alone (Envigo-Teklad TD 130343) and three mice to a fiber-free diet supplemented with SQ prebiotic (1% w/v) provided in the drinking water. Fecal pellets were collected on days 7, 13, 20, 22 and 27. DNA extractions were performed on fecal pellets with acid-washed glass beads (212 − 300 μm; Sigma-Aldrich), 500 μl Buffer A (200 mM NaCl, 200 mM Tris, 20 mM EDTA), 210 μl SDS (20% w/v, filter-sterilized) and 500 μl phenol:chloroform (1:1). The slurry was subjected to bead-beating (Biospec Products) for 3 min at room temperature then centrifuged (12,000 rpm, 4 °C, 3 min) and the aqueous phase was recovered. An equal volume of phenol:chloroform (1:1) was added and mixed by gentle inversion. After centrifugation, the aqueous phase was retained and 0.1 volume 3 M sodium acetate (pH 5.2) and 1 volume of 100% ethanol were added. The samples were mixed by gentle inversion and incubated at −80 °C for 30 min, centrifuged for 20 min (12,000 rpm, 4 °C) and the supernatants removed. The DNA pellets were washed with 70% ethanol, air-dried, and then resuspended in nuclease-free water. The resulting DNA extracts were purified by using DNeasy Blood & Tissue Kit (Qiagen). Strain abundance was quantified as described previously (31).

### Proteomics

#### Proteomic sample preparation

Independent experiments performed in triplicate were grown with glucose or sulfoquinovose, collected by centrifugation and snap frozen. Cell pellets were resuspended in lysis buffer (4% SDS, 50 mM Tris pH 8.5, 10 mM DTT) and then boiled at 95 ˚C for 10 min with shaking at 2000 rpm. Lysates were cooled to room temperature, protein concentrations were determined using a BCA assay, and 200 μg of protein for each sample was acetone precipitated by mixing 4 volumes of ice-cold acetone with one volume of sample. Samples were precipitated overnight at −20˚C, precipitated proteins were collected by centrifugation at 17000 x g for 10 min at 4 ˚C, acetone supernatant was discarded, and excess acetone was driven off at 65˚C. Dried protein pellets were resuspended in 6 M urea, and 2 M thiourea in 40 mM NH_4_HCO_3_, then reduced with 10 mM DTT for 1 h at room temperature, followed by alkylation with 40 mM iodoacetamide for 1 h. Excess iodoacetamide was quenched with 40 mM DTT and samples were digested with Lys-C (1/200 w/w, Wako Chemicals) for 4 h before being diluted by mixing with 5 volumes of 100 mM NH_4_HCO_3_, then digested with trypsin (1/50 w/w, Promega) overnight. Digested samples were acidified to a final concentration of 0.5% formic acid and desalted using C18 stage tips (44) before analysis by LC-MS.

#### Proteomics analysis using reversed-phase LC-MS

Purified peptides were re-suspended in Buffer A∗ (2% ACN, 0.1% TFA) and separated using a two-column chromatography setup composed of a PepMap100 C18 20 mm × 75 μm trap and a PepMap C18 500 mm × 75 μm analytical column (Thermo Fisher Scientific). Samples were concentrated onto the trap column at 5 μl/min for 5 min and infused into an Orbitrap Elite (Thermo Fisher Scientific). 120-min gradients were run altering the buffer composition from 3% buffer B (80% acetonitrile, 0.1% formic acid) to 26% B over 90 min, then from 26% B to 35% B over 10 min, then from 35% B to 80% B over 2 min, the composition was held at 80% B for 3 min, and then dropped to 3% B over 5 min and held at 3% B for another 10 min. The Elite Orbitrap Mass Spectrometer was operated in a data-dependent mode automatically switching between the acquisition of a single Orbitrap MS scan (300–1600 m/z; 120,000 resolution) and a maximum of 10 ion trap MS-MS scans (Rapid MS/MS mode, CID NCE 30, maximum fill time 100 ms, AGC 1∗10^4^).

#### Mass spectrometry data analysis

Proteomic analysis was accomplished using MaxQuant (v1.5.3.30) (45). Searches were performed against the *E*. *rectale* proteome (Uniprot accession: UP000001477, downloaded 19–02–2019, 3545 entries) with carbamidomethylation of cysteine set as a fixed modification. Searches were performed with Trypsin cleavage specificity allowing 2 mis-cleavage events and the variable modification of oxidation of methionine. The precursor mass tolerance was set to 20 parts-per-million (ppm) for the first search and 7 ppm for the main search, with a maximum false discovery rate of 1.0% set for protein and peptide identifications. To enhance the identification of peptides between samples the Match Between Run option was enabled with a precursor match window set to 2 min and an alignment window of 10 min. For label-free quantitation, the MaxLFQ option within Maxquant (46) was enabled. The resulting peptide outputs were processed within the Perseus (v1.4.0.6) (47) analysis environment to remove reverse matches and common protein contaminates with missing values imputed. Proteomics data and the associated search outputs have been deposited to the ProteomeXchange Consortium *via* the PRIDE partner repository with the dataset identifier: PXD053947.

### Cloning, expression, and purification of target proteins

#### *E*. *rectale* SftG and SftX

Genes encoding SftG and SftX from *E*. *rectale* were codon optimization for expression in *E*. *coli* then synthesized and cloned into the pET29a(+) expression vector (Genscript). *E*. *coli* NEB T7 Express cells were transformed with pET29-SftG or pET29-SftX cloned with C-terminal His6-tags and grown in LB media with shaking (200 rpm) at 37 °C (50 μg mL^–1^ kanamycin) until the culture reached an A_600_ of 0.8. The culture was cooled to 18 °C and isopropyl *β*-thio-D-galactopyranoside (IPTG) was added to a final concentration of 100 μM and shaking (200 rpm) continued at this temperature for 16 h. Cells were harvested by centrifugation (17,000 x g, 20 min, 4 °C), resuspended in PBS (50 mM sodium phosphate, 150 mM NaCl, pH 7.5) with a cocktail of protease inhibitors (Roche cOmplete, EDTA-free), lysed by sonication, and clarified by centrifugation (17,000 x g, 20 min, 4 °C). The supernatant was filtered (0.22 μm) and subjected to immobilized metal-ion affinity chromatography (IMAC) on a nickel nitrilotriacetic acid (Ni-NTA) column. Fractions containing the product (as determined by SDS-PAGE) were combined and further purified by size exclusion chromatography (GE Superdex 200 16/600) using PBS as a buffer. The protein obtained was estimated to be >95% pure by Coomassie-stained SDS-PAGE. Protein concentration was determined by bicinchoninic acid (BCA) assay. The yield of SftG was ≈ 50 mg L^–1^ and for SftX it was ≈ 10 mg L–1.

#### *B*. *megaterium* SqvD

A gene encoding SqvD from *Bacillus megaterium* was codon optimized for expression in *E*. *coli* then synthesized and cloned into the pET28a(+) expression vector (Table S1). Protein over-expression for SqvD was carried out in *E*. *coli* BL21 (DE3) cells using the following procedure with selenomethionine (SeMet) incorporated by metabolic inhibition using standard procedures (48). The plasmids containing the BmDUF4867 gene were used to transform *E*. *coli* BL21(DE3) competent cells for gene expression. Starter cultures were grown in LB-medium (5 ml) containing 30 μg mL^-1^ kanamycin for 18 h at 37 °C with shaking at 220 r.p.m. 1 L volume cultures were inoculated with the starter culture (5 ml) and incubated at 37°C with shaking at 220 r.p.m. until an OD_600_ of 0.6 to 0.8 was reached. Gene expression was induced by the addition of IPTG (0.5–1 mM) and shaking was continued overnight at 16 °C with shaking at 200 r.p.m. The cells were then harvested by centrifugation at 5000*g* for 20 min and resuspended in 50 mM Tris buffer pH 7.5, containing 300 mM NaCl and 30 mM imidazole. Cells were disrupted using a high-pressure cell homogenizer at 20k psi, and the suspension was centrifuged at 50,000 g for 30 min to yield a clear lysate. The N-terminal His_6_-tagged protein was purified using IMAC on a Ni-NTA column, followed by size exclusion chromatography (SEC). For IMAC, the lysate was loaded onto a pre-equilibrated Ni-NTA column, followed by washing with a load buffer (50 mM Tris, 300 mM NaCl, 30 mM imidazole pH 7.5). The bound protein was eluted using a linear gradient with a buffer containing 300 mM imidazole. Protein fractions were pooled, concentrated and loaded onto a HiLoad 16/600 Superdex 75 gel filtration column pre-equilibrated with 50 mM Tris, 300 mM NaCl pH 7.5 buffer. The protein was concentrated using a Vivaspin centrifugal concentration with 3 kDa molecular weight cut-off to a final concentration of 40 to 60 mg mL^-1^ for crystallization experiments. The yield was 40 mg L^-1^ after the two-step purification. The purity and homogeneity of protein were assessed by sodium dodecyl sulfate-polyacrylamide gel electrophoresis (SDS-PAGE) and SEC-MALLS analysis, and protein concentration was determined by bicinchoninic acid assay. For SDS-PAGE, the protein samples were prepared with SDS and reducing agent (2-mercaptoethanol), heat-denatured at 95 °C for 10 min, and loaded onto a 4 to 20% Mini-PROTEAN TGX precast protein gel (Bio-Rad) with a broad-range marker ladder (15–100 kDa). The proteins were separated based on their charge and size by electrophoresis run at a constant voltage of 200V for 30 min. The gel was stained with Coomassie Brilliant Blue G-250 (ThermoFisher Scientific) for visualization.

### *E*. *rectale* SftG kinetics

#### Michaelis-Menten kinetics

Michaelis-Menten kinetics were determined in PBS (50 mM sodium phosphate, 150 mM NaCl, pH 6.5). Reactions were incubated at 30 °C and monitored at 399 nm. The concentrations of PNPSQ used were: 0.1, 0.2, 0.5, 0.8, 1.0, 1.5, 2.0, 3.0, 4.0, and 5.0 mM. Reactions were initiated by the addition of 5 μl of SftG to obtain a final volume of 800 μl and a final enzyme concentration of 7.14 × 10^-6^ mM. Initial rates were extracted and plotted as Michaelis-Menten and Lineweaver Burk plots using Prism 5.

#### pH profile

For the pH profile *k*_cat_/*K*_M_ values were calculated using the substrate depletion method. Individual cuvettes containing 800 μl (final volume) of 0.03 mM PNPSQ were prepared in different pH buffers comprised of citric acid 50 mM, Na_2_HPO_4_ 50 mM, and 150 mM of NaCl. The reactions were initiated by the addition of 5 μl of SftG such that the final enzyme concentration was 7.14 × 10^-6^ mM. The reaction was monitored at 399 nm for 60 min. The data was fit with an exponential function using Prism 5 to obtain *k*_cat_/*K*_M_ values.

### Investigation of activities for *E*. *rectale* SftX

#### Mutarotase

Separate reactions were carried out with 10 mM solutions of SQ, SF, and SR made up in sodium phosphate buffer [50 mM] with NaCl [150 mM] (pH = 7.0, total volume of 500 μl). Enzymatic reactions were initiated by the addition of *E*. *rectale* SftX, so that the final reaction concentration of the protein was 4.4 μM. The samples were incubated at 37 °C for 24 h in sterile Eppendorf tubes. After 24 h, the protein was deactivated by heating at 80 °C for 3 min followed by evaporating to dryness and further drying under a high vacuum. The dried samples were dissolved in D_2_O and analysed using ^1^H EXSY NMR (700 MHz) spectroscopy, these samples were analyzed through comparison with known standards and showed no evidence of mutarotase function. Incremental increases in the concentration of DUF protein did not affect ^1^H EXSY spectrum.

#### Kinase

Reactions were carried out with a 10 mM solution of SF in bis-tris-propane (BTP) buffer [25 mM] with KCl [25 mM], MgCl_2_ [5 mM] (pH = 7.5, total volume of 500 μl) with ATP [5 mM] and 1 mg mL^-1^ BSA. Enzymatic reactions were initiated by the addition of *E*. *rectale* SftX, so that the final reaction concentration of the protein was 4.4 μM. The sample was incubated at 37 °C for 24 h in sterile Eppendorf tubes. After 24 h, the protein was deactivated by heating at 80 °C for 3 min. The sample was analyzed using a Thermo OrbiTrap Exactive mass spectrometer, through comparison with known standards of SF and SFP, and showed no evidence of kinase function. The addition of supplementary amounts of SftX protein had no effect.

### SEC-MALLS analysis

Experiments were conducted on a system comprising a Wyatt HELEOS-II multi-angle light scattering detector and a Wyatt rEX refractive index detector linked to a Shimadzu HPLC system (SPD-20A UV detector, LC20-AD isocratic pump system, DGU-20A3 degasser and SIL-20A autosampler). Work was conducted at room temperature (20 ± 2°C). The solvent was 0.2 μm filtered before use and a further 0.1 μm filter was present in the flow path. The column was equilibrated with at least 2 column volumes of solvent before use and flow was continued at the working flow rate until baselines for UV, light scattering and refractive index detectors were all stable. Sample injection volume was 100 μl at 2.7 mg ^mL-1^ concentration; Shimadzu LabSolutions software was used to control the HPLC and Astra 7 software for the HELEOS-II and rEX detectors. The Astra data collection was 1 min shorter than the LC solutions run to maintain synchronization. Blank buffer injections were used as appropriate to check for carry-over between sample runs. Data were analyzed using the Astra 7 software. MWs were estimated using the Zimm fit method with degree 1. A value of 0.182 was used for protein refractive index increment (dn/dc). The running buffer was 50 mM Tris pH 7.5, 300 mM NaCl.

### NanoDSF analysis

NanoDSF studies were performed on a Prometheus NT.48 (NanoTemper). Data recording and initial analysis were performed with PR.ThermControl software. DUF4867 samples were at 1 mg mL^-1^ in 50 mM Tris, 300 mM NaCl pH 7.5. and pre-incubated with 5 mM ligand at 25 °C. All samples were centrifuged at 13,000 rpm for 2 min prior to loading and 15 μl was loaded onto the capillary per sample. Experiments were performed in duplicates with the temperature ramp from 25 °C to 95 °C, at 1.0 °C/min with 45% excitation power.

### Inductively coupled plasma optical emission spectroscopy (ICP-OES) analysis

Samples of protein and buffer blank (100 μl sample solution as supplied) were aliquoted into acid-leached Teflon digestion tubes with washing (2 x 100 μl Milli-Q 18.2 MΩ ultrapure water system supplied by Merck). Aqueous HCl (1.00 ml, 37%, certified AR grade, supplied by Fisher Chemical) and HNO_3_ (3.00 ml, 70%, certified AR grade, supplied by Fisher Chemical) were added. Samples were digested in an Anton-Paar Multiwave Go Plus with microwave irradiation, ramp rate 18 °C min^-1^, ultimate temperature 180 °C, dwell time 15 min. Samples were allowed to cool and diluted to final volume (50 ml) in grade A acid-leached glassware with ultrapure water. No turbidity was noted in the final samples. A digestion recovery test solution was prepared in the same manner as the above samples (1.00 ml 10 ppm working reference), as well as a digestion blank (1.00 ml ultrapure water). Working standard solutions were prepared from a commercial reference standard CCS-6 supplied by Inorganic Ventures, traceable to NIST-certified reference materials. All working standards were matrix-matched to the digestion media. ICP-OES was conducted using an Agilent ICP-OES 5800 VDV spectrometer. Cd (228.802 nm), Co (238.892 nm), Cr (205.560 nm), Cu (327.395 nm), Fe (259.940 nm), Mn (259.372 nm), Ni (221.648 nm), Pb (283.305 nm), Tl (190.794 nm), V (292.401 nm), and Zn (206.200 nm) analyses were conducted with internal standard Y (371.029 nm). Measurements were made in axial configuration, plasma flow 12.0 L min^-1^, auxiliary flow 1.00 L min^-1^, radiofrequency power 1.20 kW. The limit of detection (LOD) and limit of quantification (LOQ) were defined as 3 and 10 times the standard deviation of the concentration of the calibration blank respectively. The exact values for each element are provided in the supplemental information.

### Crystallization and structure determination of the SftG⋅ IFGSQ complex

A single crystal was grown over 3 days in sitting drops at 20 °C by mixing 1 μl well solution containing 15% PEG 4000, 0.1 M MgCl_2_, 0.05 M Tris-HCl, pH 8.0, with 1 μl SftG solution at 6 mg mL^-1^. The aza-sugar inhibitor IFGSQ was soaked into the crystal overnight using 2 μl of 10 mM IFGSQ in mother liquor. The crystal was cryo-protected by supplementing the mother liquor with 3 M ammonium sulfate and was cryo-cooled using liquid nitrogen. Data was collected at the Australian Synchrotron (MX2 beamline) and processed using XDS (49). The structure was solved by molecular replacement using PHASER (50) and the sulfoquinovosidase YihQ from *E*. *coli* as a search model (PDB ID: 5AED) (23). The final model was built in Coot (51) and refined with Phenix (52) to a resolution of 1.90 Å. Data collection and refinement statistics are summarized in Table S2. The R_work_ and R_free_ after the final refinement were 0.1763 and 0.2170, respectively. Figures were prepared using Pymol. The coordinate files and structure factors have been deposited in the Protein Data Bank (PDB) with accession code **6PNR**.

### Initial screening and optimized crystallization conditions for *B*. *megaterium* SqvD

Initial screening was performed using commercially available INDEX, Crystal HT, PEG/ion HT (from (Hampton Research), and PACT premier (Molecular Dimensions) screens in 96-well sitting drop trays. Further optimization was carried out in a 48-well sitting drop or 24-well hanging-drop format to obtain optimal crystals for X-ray diffraction.

A crystal of ligand-free *B*. *megaterium* SqvD was grown using a 60 mg mL^-1^ protein solution in 50 mM Tris buffer pH 7.5 containing 300 mM NaCl in a drop with 0.15 μl protein: 0.15 μl mother liquor, the latter comprising 2.0 M sodium chloride and 10% w/v PEG (polyethylene glycol) 6000. A crystal of selenomet_SqvD grew from 40 mg mL^-1^ enzyme in 50 mM Tris buffer pH 7.5 containing 300 mM NaCl, in a drop with 0.1 μl protein: 0.2 μl mother liquor, with the reservoir solution containing 2.0 M ammonium sulfate and 5% v/v 2-propanol. SqvD⋅SF complex was achieved using direct soaking of SF ligand into a crystal of SqvD, which was obtained from protein solution at 60 mg mL^-1^ in 50 mM Tris buffer, 300 mM NaCl buffer pH 7.5 used to set up a drop containing 0.15 μl protein: 0.15 μl mother liquor, the latter comprising 0.1 M HEPES sodium 7.5 30% v/v polyethylene glycol 400. All crystals were harvested into liquid nitrogen, using nylon CryoLoops (Hampton Research) using mother liquor without any cryoprotectants.

### Data collection, processing, and refinement for *B*. *megaterium* SqvD

Data were processed and integrated using XDS (49) and scaled using SCALA (53) included in the Xia2 processing system (54, 55). Data collection and refinement statistics are given in Table S3. The initial structure was solved using single-wavelength anomalous dispersion (SAD) methods, at the f″ peak wavelength (0.9797 Å, judged from the X-ray fluorescence scan), from the Se-Met derivative of the protein through automated pipelines (BigEP) at diamond comprising substructure search and experimental phasing using SHELXC/D/E (56). Early model building was automated using AUTOBUILD (57). Native and SF-soaked structures were solved using MOLREP (58), SeMet SqvD as an initial search model. The structure was built and refined using iterative cycles using COOT (51) and REFMAC (59), the latter employing local NCS restraints. Following building and refinement of the protein and water molecules, clear residual density was observed in the omit maps for metal and ligands. The coordinate and refinement library files for ligands (SF) were prepared using ACEDRG (60). Metal was modeled at occupancy of 0.8 to 0.9. All steps were performed from within the CCP4i2 suite (61). The coordinate files and structure factors have been deposited in the Protein DataBank (PDB) with accession codes **9GYY** and **9GYZ**.

### DUF4867 family sequence analysis

DUF4867 protein sequences were retrieved from Interpro family IPR032358. The retrieved sequences were used to generate a sequence similarity network (SSN) at different alignment scores using EFI-EST tool (https://efi.igb.illinois.edu/efi-est/) (27). The SSN generated at an alignment score of 70 with representative nodes of 100% identity was used to generate genome neighborhood network (GNN) and genome neighborhood diagrams (GNDs) with open reading frame (ORF) ± 10 neighbors using the Enzyme Function Initiative-Genome Neighbourhood Tool (https://efi.igb.illinois.edu/efi-gnt/). The individual nodes of the DUF4867 SSN were manually colored in Cytoscape (62) based on the occurrence of gene homologs from proposed pathways in their neighborhood. Also, the nodes of SSN were manually colored based on their phylum.

## Data availability

Supporting information includes Figures S1-S8, Tables S1-S3, a list of accession codes for DUF4867 homologs, and SSN and GNN tables (Excel spreadsheet).

Proteomics data and the associated search outputs have been deposited to the ProteomeXchange Consortium *via* the PRIDE partner repository with the dataset identifier: PXD053947.

X-ray crystallography data is available at the Protein Databank archive (https://www.rcsb.org/) under the accession codes 6PNR, 9GYY, and 9GYZ.

## Supporting information

This article contains supporting information.

## Conflict of interest

The authors declare that they have no conflicts of interest with the contents of this article.
