# Supplementary material for: Sulfoglycolysis sustains Eubacterium rectale in low-fiber diets
Source: J Biol Chem. 2025 Feb 14;301(3):108320. doi: 10.1016/j.jbc.2025.108320 (PMC11968277; doi:10.1016/j.jbc.2025.108320)
Supplement: Supplementary Material 1 [file mmc1.docx]

**Sulfoglycolysis sustains *Eubacterium rectale* in low-fiber diets**

Mahima Sharma,^1^ Nicholas Pudlo,^2^ Michael A. Järvå,^3,4^ Arashdeep Kaur,^5^ Alan John,^3,4^ Laura Burchill,^5^ James P. Lingford,^3,4^ Ruwan Epa,^5^ Palika Abayakoon,^5^ Nichollas E. Scott,^6^ Johan P. Turkenburg,^1^ Gideon J. Davies,^1^* Eric C. Martens,^2^* Ethan D. Goddard-Borger,^3,4^* Spencer J. Williams^5^*

1. **Supplementary Figures**

**
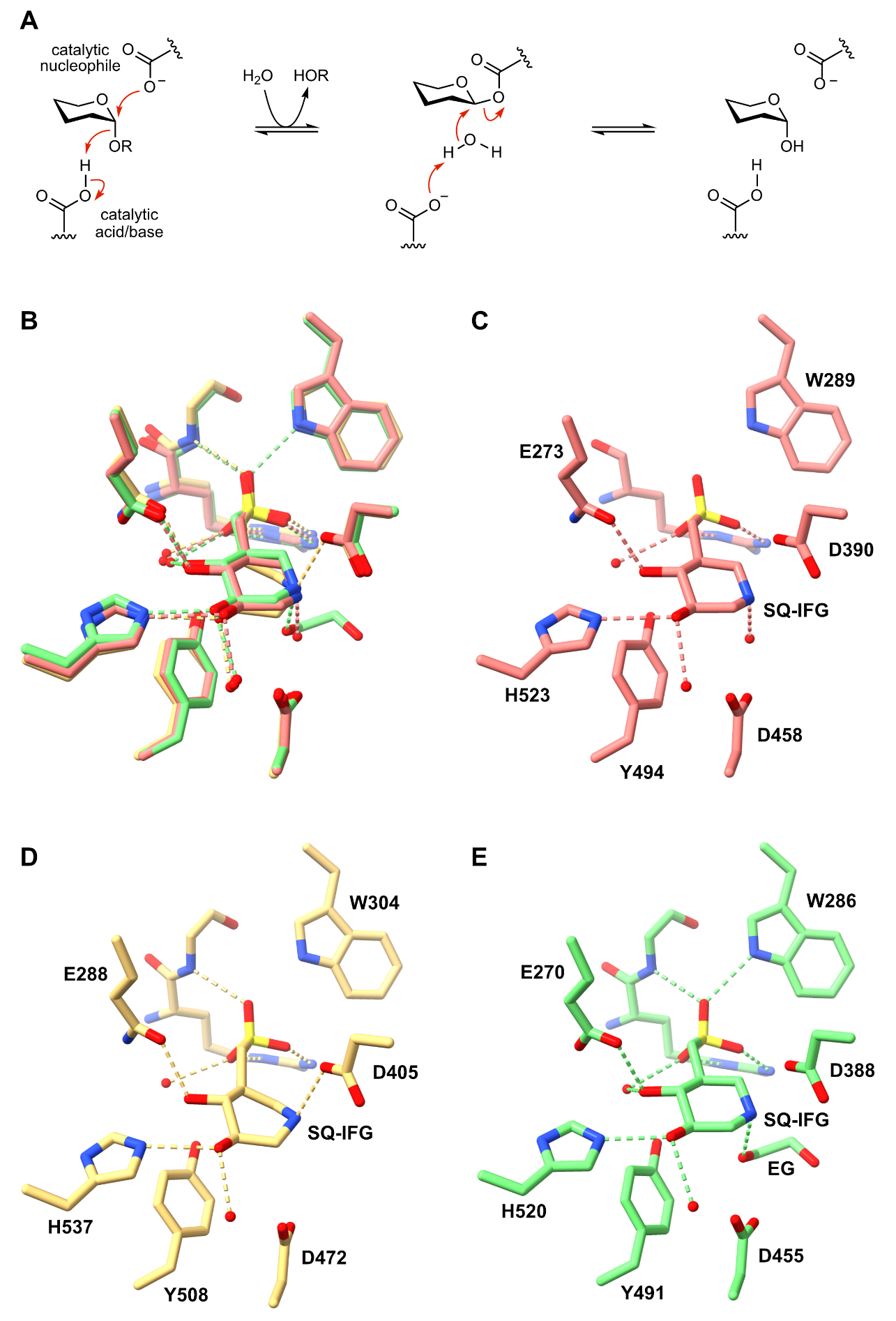
**

**Figure S1.** (a) Retaining GH31 SQases use a double-displacement mechanism involving a catalytic acid/base and nucleophile residue. (b) Overlay of the active sites of *E. rectale* SftG (pink, pdb 6PNR), *E. coli* YihQ (yellow, pdb 5OHT) and *A. tumefaciens* SQase (green, pdb 5OHY) with bound SQ-IFG. (c-e) Individual active sites with residues and ligands labelled for clarity (note: ‘EG’ is ethylene glycol cryoprotectant).


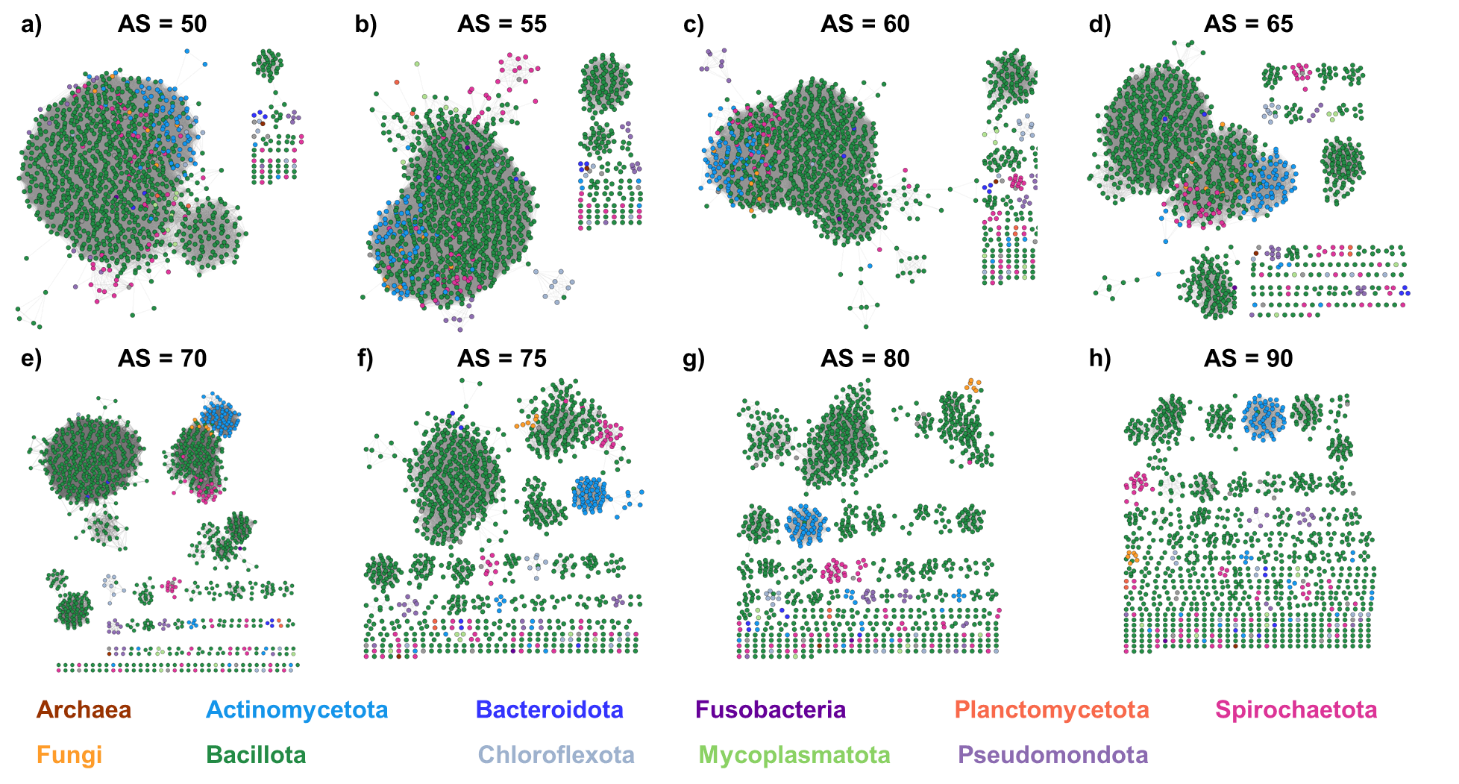


**Figure S2.** **Sequence similarity network of DUF4867 family members at different minimum alignment scores.** (a) SSN at minimum alignment score of 50, (b) SSN at minimum alignment score of 55, (c) SSN at minimum alignment score of 60, (d) SSN at minimum alignment score of 65, (e) SSN at minimum alignment score of 70, (f) SSN at minimum alignment score of 75, (g) SSN at minimum alignment score of 80, and (h) SSN at minimum alignment score of 90. Nodes are colored based on taxonomy.


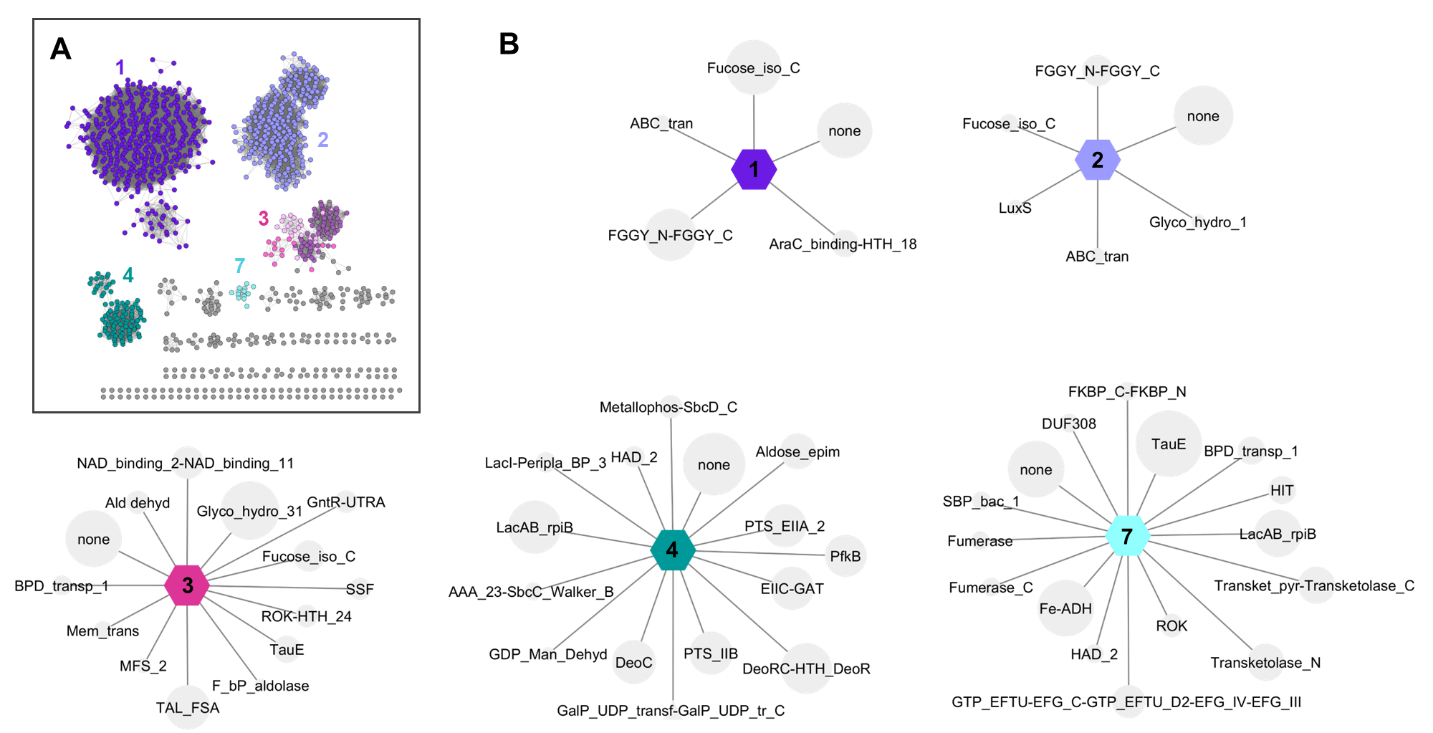


**Figure S3.** **Genome neighborhood network (GNN) of sequence similarity network (SSN) of DUF4867 proteins.** (a) SSN of DUF4867 protein members at minimum alignment score of 70. (b) GNN of SSN of DUF proteins at alignment score of 70. Hub nodes are the SSN cluster number and spoke nodes are the neighborhood protein families (Pfam).

**
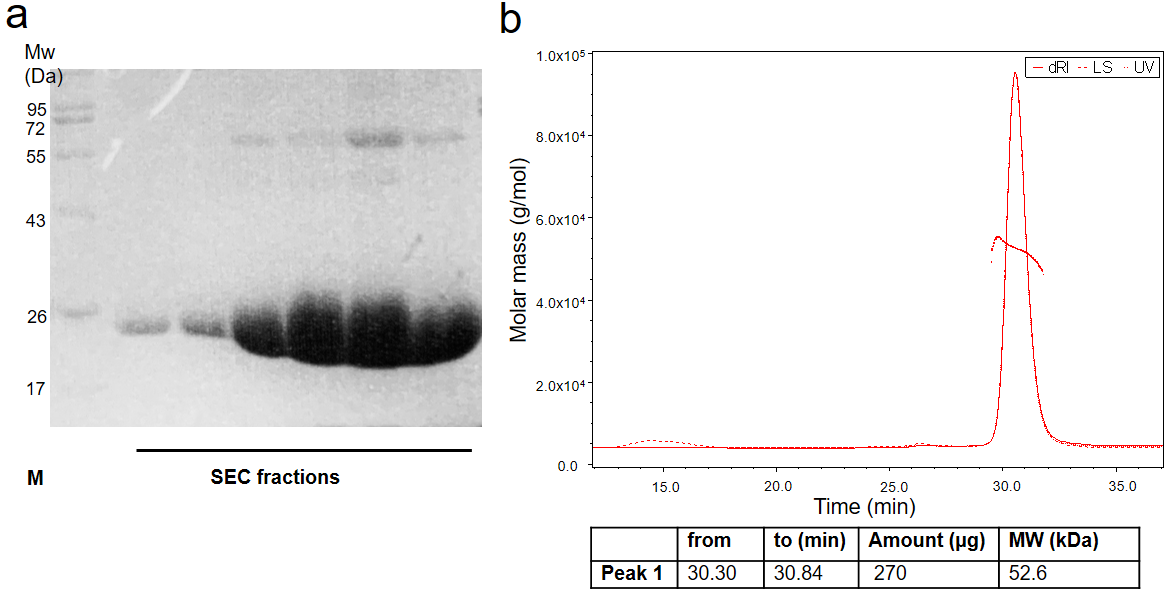
**

**Figure S4**. **Purification and molecular assembly of DUF4867.** (a) Coomassie-stained SDS-PAGE analysis of purified DUF4867 protein SqvD from *Bacillus megaterium* after IMAC and size exclusion chromatography (SEC) showing expected MW of ~26,000 Da. (b) SEC-MALLS molar mass plot reveals the oligomeric state of SqvD in solution. UV-trace and an average molecular weight trace (red), calculated from the refractive index and light scattering signal gave a mass estimate of 53 kDa, which corresponds to a dimer and comprises >99% of the eluted material, confirming homogeneity of the sample. Note: empty gel lanes (to the right) or containing protein samples unrelated to this project have been cropped off for clarity.


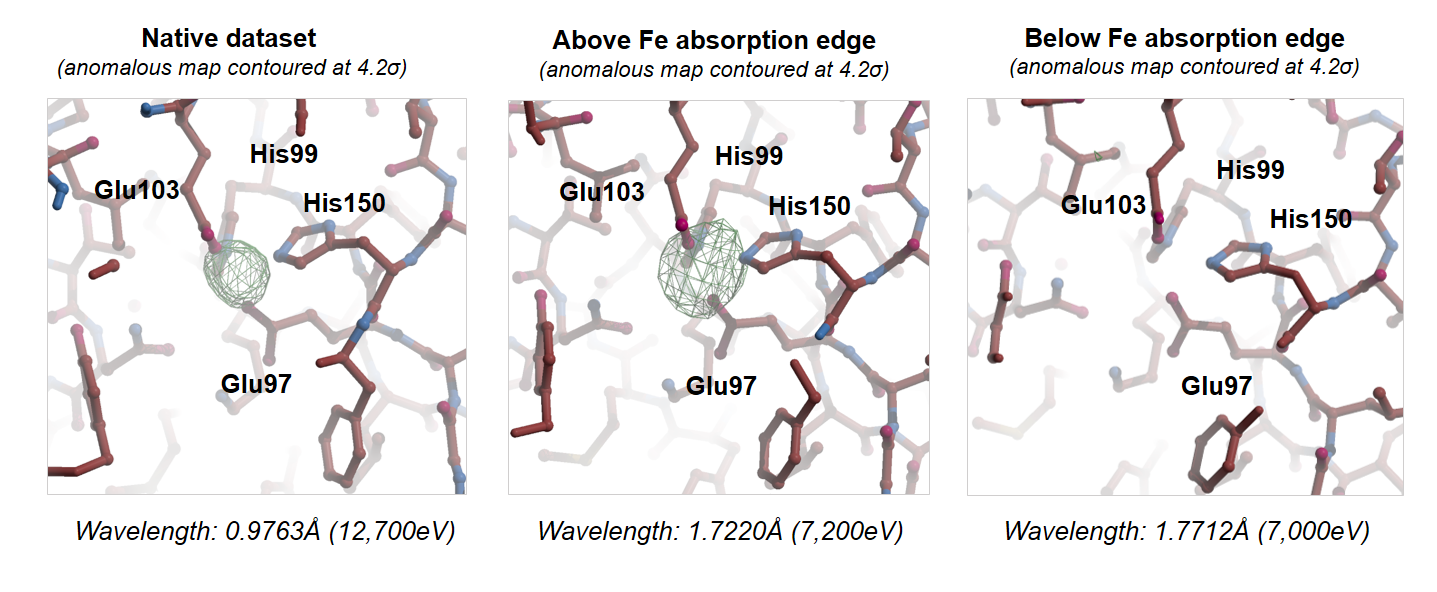


**Figure S5.** Comparison of anomalous difference Fourier maps (contoured at 4.2 σ). Fe was identified and modelled in the native crystal structure. Above the edge (middle), the measured anomalous signal specific to Fe was observed, whereas below the edge (right), the anomalous signal was negligible.


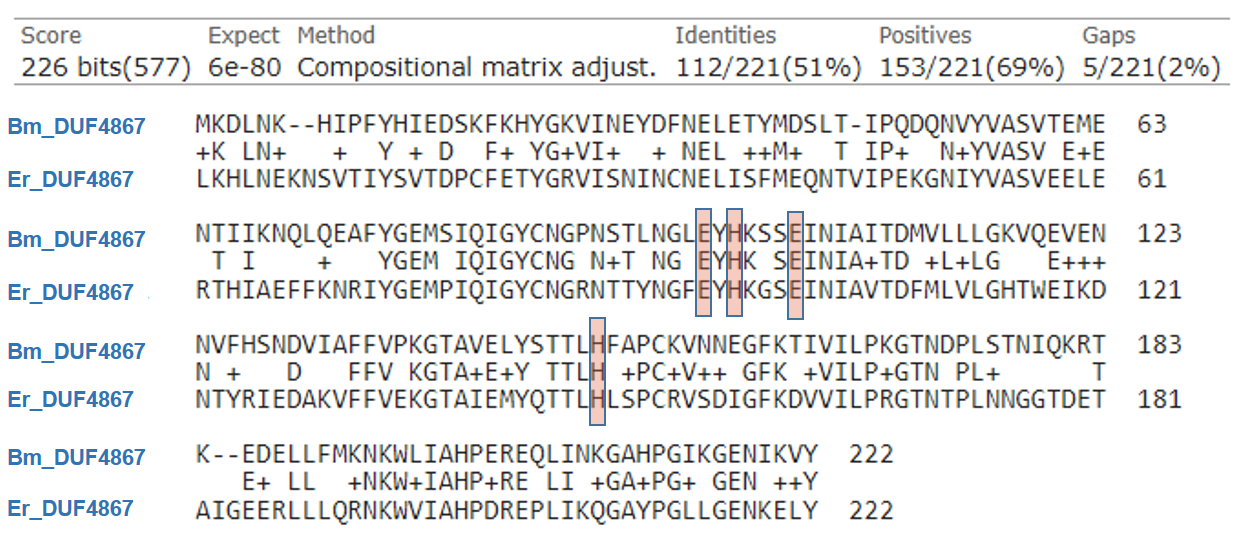


**Figure S6. DUF4867 proteins share cupin-type fold with conserved 2His-2Glu metal binding motif.** Sequence alignment of DUF4867 proteins from *Eubacterium rectale* (SftX) and *Bacillus megaterium* (SqvD). All four metal binding residues are highlighted in red.

**
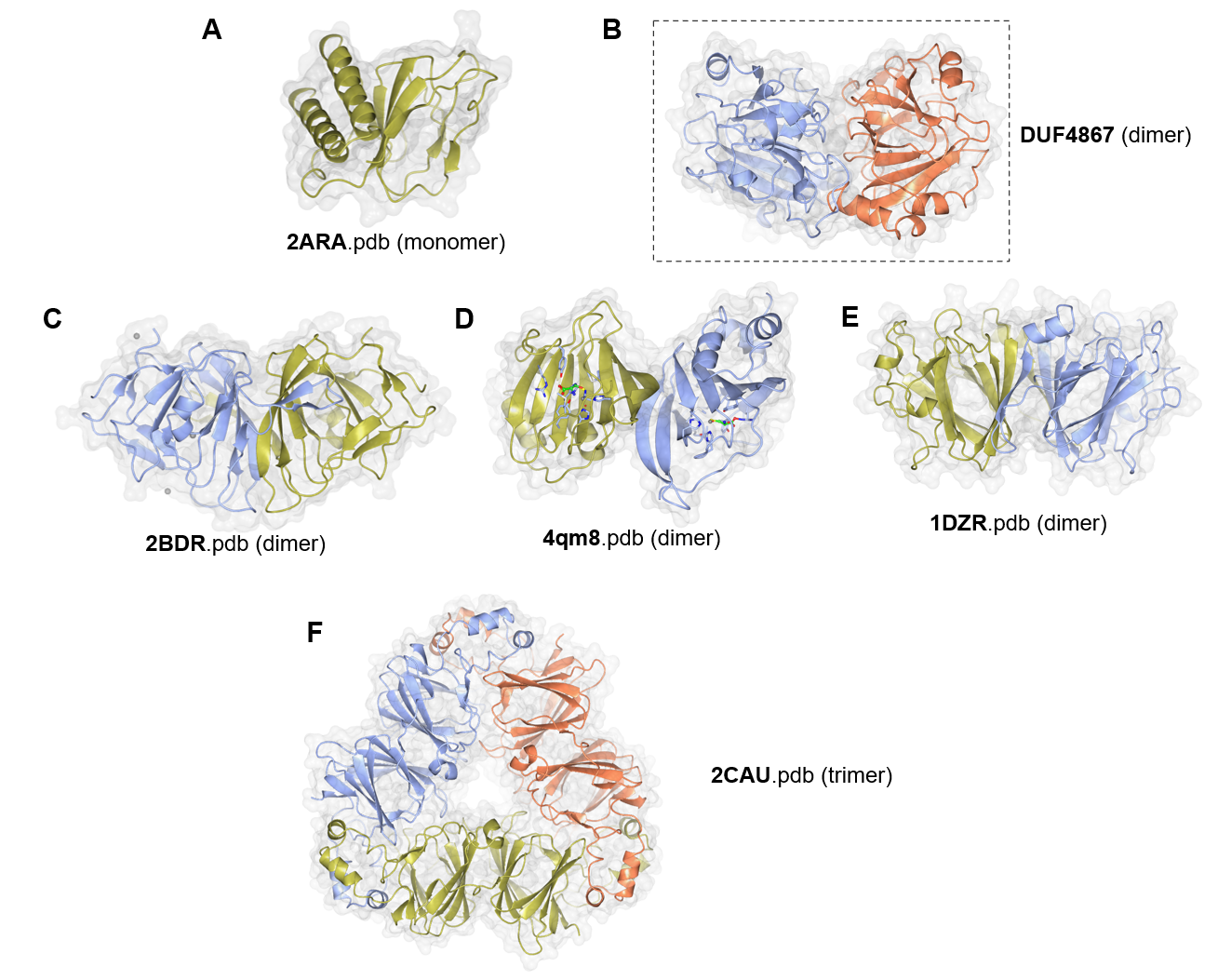
**

**Figure S7.** **Oligomeric states and functions of 3D structures of cupin family proteins.** (A) arabinose-binding domain of AraC transcription factor (PDB code: 2ARA) from *E. coli*. (B) *Bacillus megaterium* DUF4867 protein SqvD. (C) Putative ureidoglycolate hydrolase PP4288 from *Pseudomonas putida* (2BDR). (D) Putative cysteine dioxygenase from *Bacillus subtilis*. (E) dTDP-4-dehydrorhamnose 3,5-epimerase RmlC from *Salmonella enterica* (1DZR) exists as dimer in solution. (F) Structure of two-domain canavalin from *Canavalia ensiformis* (jack bean) (2CAU) that forms a trimer in solution.


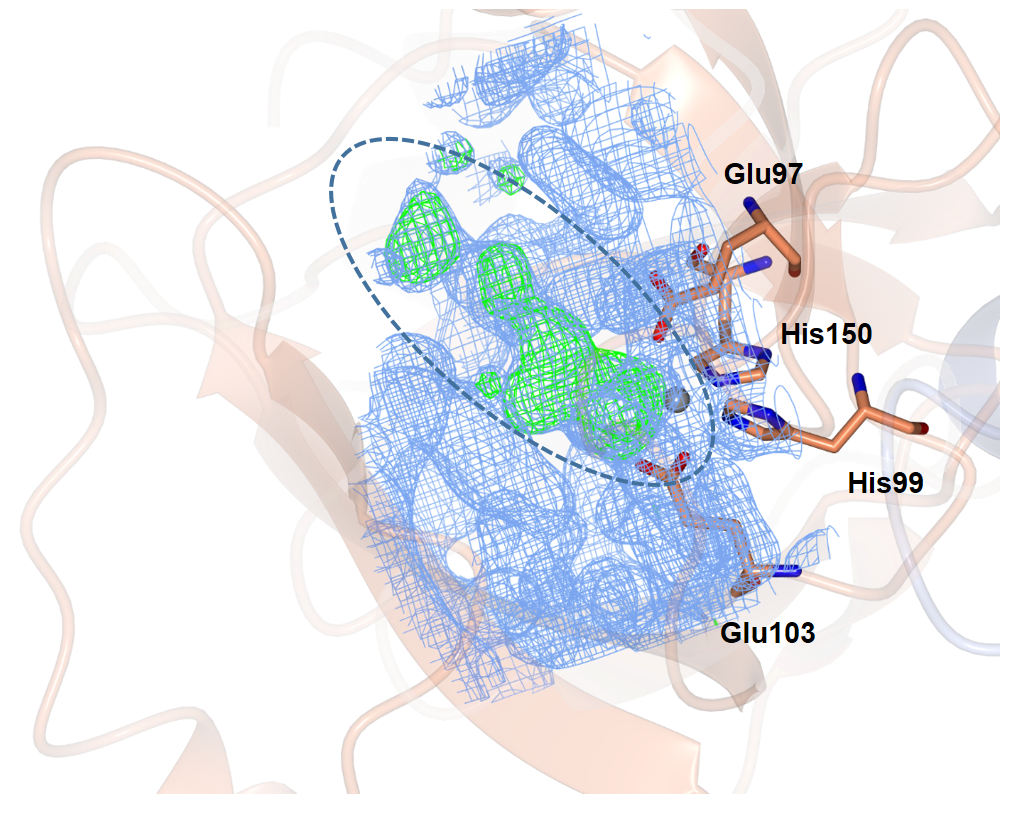


**Figure S8.** **Close up view of structure of ligand-bound SqvD**. Data obtained upon soaking of crystal of SqvD with SF. Electron density corresponds to Fo-Fc omit map (in green) at levels of 2.5σ, generated after modelling inclusion of the metal and solute, showing additional density of a ligand bound to the metal.


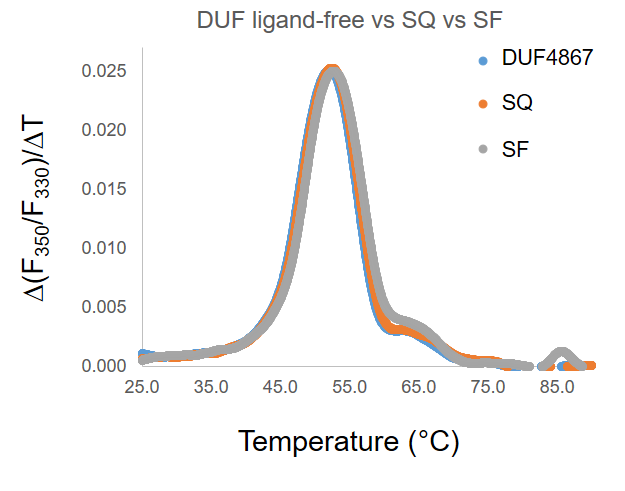


**Figure S9. NanoDSF Bacillus megaterium SqvD.** Thermal unfolding profile showing no shift in melting temperature (Tm) of ligand-free protein (blue), versus when incubated with 10 mM SQ (orange) or SF (grey).

**Figure S10. Proposed mechanisms of ureidoglycoate lyase, KdgF and DUF4867 (functioning as a ring-opening isomerase).** a) proposed mechanism of ureidoglycolate lyase, b) proposed mechanism of KdgF unsaturated-uronate lyase, c) proposed mechanism of DUF4867 if acting as ring-opening isomerase.**Supplementary Tables**

**Table S1.** Amino acid sequence of recombinant *E. rectale* SftG and SftX, and *B. megaterium* SqvD

*E. rectale* SftG

MQIKVNDNEFQLFVGEKRILEHSKERPMIYVGVGQEDVDMYRGNFKITDYVTERFPLKLTDVIQTADTVRLCFESYIIAKIKCDENLCTIDFEQKDDRINRFWFRVAADKEEKCYGCGEQMSYFNLRGRNFPIWTSEPGVGRDKTTYVTWRSDVENKAGGDYYNTNYPQPTFVSTNKYYLHVDSTAYADFDFRNDSFHELQIWEVPKQIRIECADTYLKLLERITTYFGRQPKLPDWVYNGLIIGVQGGNERSFGLLDKTLDRNIKVAGIWCQDWCGKRVTSFGKRLQWDWKYHKEMYPDLPKKIKEINAKGIKFLGYVNPYLVNDGELYKEGKEKGYFATKADGSDYLVDFGEFYCGVVDLTNPEAFEWFKDIIKEYTLGIGIDGWMADFGEYLPTDDICLYSGKSPMIEHNHWPVLWAKCNYEAVKESGKLGDVVYFMRAGGAGSQKYCTLLWAGDQSVDFTIHDGLASVICGALSAGMMGCGLTHSDIGGYTSLFDNTRTKELFLRWAEMAMFTPFMRTHEGNRPDTNFQYYDDEDTMERLARLVDVYTMLAPYTKTLVEENADSGHPVQRPLFMHYESDAKAYDIQYEYLFGRDMLIAPVYEQDKHEWDVYLPQDEWVHLWTGEEYHGGEITVSAELGYTPAFYRKNSEFADIFEEIREKYGVKLEHHHHHH

*E. rectale* SftX

MLKHLNEKNSVTIYSVTDPCFETYGRVISNINCNELISFMEQNTVIPEKGNIYVASVEELERTHIAEFFKNRIYGEMPIQIGYCNGRNTTYNGFEYHKGSEINIAVTDFMLVLGHTWEIKDNTYRIEDAKVFFVEKGTAIEMYQTTLHLSPCRVSDIGFKDVVILPRGTNTPLNNGGTDETAIGEERLLLQRNKWVIAHPDREPLIKQGAYPGLLGENKELYYLEHHHHHH

*B. megaterium* SqvD

MGSSHHHHHHSSGLVPRGSHMGSFQYMKDLNKHIPFYHIEDSKFKHYGKVINEYDFNELETYMDSLTIPQDQNVYVASVTEMENTIIKNQLQEAFYGEMSIQIGYCNGPNSTLNGLEYHKSSEINIAITDMVLLLGKVQEVENNVFHSNDVIAFFVPKGTAVELYSTTLHFAPCKVNNEGFKTIVILPKGTNDPLSTNIQKRTKEDELLFMKNKWLIAHPEREQLINKGAHPGIKGENIKVYQ*

[thrombin cleavage site (underlined) with the cleaved part is highlighted in grey]

**Table S2.** Data collection and refinement statistics for SftG.

| ***Data collection*** |  |
| --- | --- |
| Space group | P 2_1_ 2_1_ 2_1_ |
| No of molecules in AU | 2 |
| ***Cell dimensions*** |  |
| *a*, *b*, *c* (Å) | 102.62, 122.67, 126.73 |
| α, β, γ (°) | 90.00, 90.00, 90.00 |
| Wavelength (Å) | 0.9537 |
| Resolution (Å)* | 49.36-1.90 (1.93-1.90) |
| *R*_sym_ or *R*_merge_* | 0.081 (1.334) |
| *R*_pim_* | 0.059 (1.052) |
| *I* / σ*I** | 10.3 (1.0) |
| CC(1/2) | 0.998 (0.348) |
| Completeness (%)* | 100.0 (100.0) |
| Redundancy* | 5.3 (4.7) |
| Wilson B-factor (Å^2^) | 32.5 |
|  |  |
| ***Refinement*** |  |
| Resolution (Å) | 47.56-1.90 |
| No. reflections | 126,117 |
| *R*_work_ / *R*_free_ | 0.1763/0.2170 |
| No. non-hydrogen atoms |  |
| Protein | 1110,974 |
| IFGSQ | 26 |
| Water | 795 |
| *B*-factors |  |
| Protein | 30.48 |
| IFGSQ | 26.83 |
| Water | 38.31 |
| R.m.s. deviations |  |
| Bond lengths (Å) | 0.006 |
| Bond angle (°) | 0.809 |
| Ramachandran plot |  |
| Favored (%) | 96.39 |
| Allowed (%) | 3.61 |
| Outliers (%) | 0 |
| **PDB code** | **6PNR** |

*Values in parentheses are for highest-resolution shell.

**Table S3.** Data collection and refinement statistics. Numbers in brackets refer to data for highest resolution shells.

|  | ***Bm_*SqvD** | ***Bm_*SqvD•SF** | ***SeMet_Bm*_SqvD** |
| --- | --- | --- | --- |
| ***Data collection*** | | | |
| Space group | P3_2_21 | P3_2_21 | P3_2_21 |
| Molecules in A.S.U | 2 | 2 | 2 |
| ***Cell dimensions*** | | | |
| *a*, *b*, *c* (Å) | 83.83, 83.83, 133.93 | 82.51, 82.51, 136.19 | 83.94, 83.94, 134.55 |
| α, β, Ɣ (°) | 90.0, 90.0, 120.0 | 90.0, 90.0, 120.0 | 90.0, 90.0, 120.0 |
| Resolution (Å) | 66.97-1.50 (1.53-1.50) | 49.30-2.10 (2.16-2.10) | 44.85-2.30 (2.38-2.30) |
| *R*_merge_ | 0.055 (1.103) | 0.101 (1.323) | 0.125 (1.137) |
| *R*_pim_ | 0.013 (0.247) | 0.025 (0.313) | 0.041 (0.372) |
| *I* / σ*I* | 28.1 (3.1) | 17.2 (2.0) | 15.5 (3.2) |
| CC1/2 | 0.999 (0.881) | 0.999 (0.905) | 0.999 (0.889) |
| Completeness (%) | 100.0 (100.0) | 100.0 (100.0) | 100.0 (100.0) |
| Redundancy | 20.5 (20.8) | 16.7 (18.6) | 19.4 (19.8) |
| No. unique reflections | 87735 (4303) | 32018 (2585) | 25035 (2400) |
| ***Refinement*** | | | |
| Resolution (Å) | 66.90-1.5 | 49.30-2.10 | *Not refined* |
| *R*_work_ / *R*_free_ | 0.1760/0.1980 | 0.2080/0.2550 |  |
| No. of atoms | |  |  |
| Protein | 3567 | 3494 |  |
| Ligand/ion | 2 (Fe) | 2 (Fe) |  |
| Water | 387 | 102 |  |
| *B*-factors (Å^2^) | | | |
| Protein | 23 | 49 |  |
| Ligand/ion | 18 (Fe) | 51 (Fe) |  |
| Water | 33 | 45 |  |
| R.m.s. deviations | | | |
| Bond lengths (Å) | 0.0119 | 0.0154 |  |
| Bond angles (°) | 2.1630 | 2.3240 |  |
| Ramachandran Plot Residues | | | |
| In most favourable regions (%) | 95.7 | 94.1 |  |
| In allowed regions (%) | 4.3 | 5.7 |  |
| Outliers (%) | 0.0 | 0.2 |  |
| *MolProbity score* | 1.51 | 1.56 |  |
| **PDB code** | **9GYY** | **9GYZ** |  |

**Table S4.** Inductively coupled plasma-optical emission spectroscopy (ICP-OES) analysis for quantitative metal detection in DUF4867 sample in solution.

|  | **Element line (concentration in ppm)** | | | | | | | |
| --- | --- | --- | --- | --- | --- | --- | --- | --- |
| **Sample** | Cd  228.802 nm  (ppm) | Co  238.892 nm  (ppm) | Cr  205.560 nm  (ppm) | Cu  327.395 nm  (ppm) | Fe  259.940 nm  (ppm) | Mn  259.372 nm  (ppm) | Ni  221.648 nm  (ppm) | Pb  283.305 nm  (ppm) |
| **DUF4867** | <LOD | <LOD | <LOD | <LOD | 8.5 | 0.65 | LOD < x < LOQ | <LOD |
| **Buffer control** | <LOD | <LOD | <LOD | <LOD | <LOD | <LOD | <LOD | <LOD |
| **Check standard** | 0.20 | 0.2007 | 0.2024 | 0.2006 | 0.1988 | 0.1995 | 0.2024 | 0.1971 |
| **Std recovery %** | 101 | 100 | 101 | 100 | 99 | 100 | 101 | 99 |

*Contd.*

|  | **Element line (concentration in ppm)** | | |
| --- | --- | --- | --- |
| **Sample** | Tl  190.794 nm  (ppm) | V  292.401 nm  (ppm) | Zn  206.200 nm  (ppm) |
| **DUF4867** | <LOD | <LOD | 2.45 |
| **Buffer control** | <LOD | <LOD | <LOD |
| **Check standard** | 0.1934 | 0.2004 | 0.206 |
| **Std recovery %** | 97 | 100 | 103 |

<LOD indicates sample below detection limit for element

LOD < X < LOQ indicates the presence of the element in the sample, but cannot be quantified above confidence limit.

**Table S5.** Glycoside hydrolases encoded by Eubacterium rectale ATCC 33656, their predicted functions and putative roles of the encoding gene clusters.

| **New locus tag** | **Old locus tag** | **accession #** | **CDS** | **CAZy**  **family** | **putative function of gene prodct** | **predicted localisation** | **predicted operon**  **(direction coordinates)** | **putative function**  **of operon** |
| --- | --- | --- | --- | --- | --- | --- | --- | --- |
| EUBREC_RS00145 | EUBREC_0030 | [ACR73835.1](https://www.ncbi.nlm.nih.gov/entrez/viewer.fcgi?db=protein&val=ACR73835.1) | WP_012740958.1 | GH18 | peptidoglycan hydrolase | unknown | 38355 - 42008 | peptidoglycan processing |
| gnpA | EUBREC_0138 | [ACR73943.1](https://www.ncbi.nlm.nih.gov/entrez/viewer.fcgi?db=protein&val=ACR73943.1) | WP_012741064.1 | GH112 | galacto-*N*-biose or lacto-*N*-biose phosphorylase | cytoplasmic | 125441 - 133125 | galacto-N-biose/lacto-N-biose catabolism |
| EUBREC_RS01165 | EUBREC_0257 | [ACR74061.1](https://www.ncbi.nlm.nih.gov/entrez/viewer.fcgi?db=protein&val=ACR74061.1) | WP_012741182.1 | GH32 | sucrose-6-phosphate hydrolase | cytoplasmic | 238151 - 251020 | sucrose catabolism |
| EUBREC_RS01270 | EUBREC_0282 | [ACR74086.1](https://www.ncbi.nlm.nih.gov/entrez/viewer.fcgi?db=protein&val=ACR74086.1) | WP_012741207.1 | GH32 | β-fructofuranosidase | cytoplasmic | 265575 - 271241 | fructan catabolism |
| EUBREC_RS02210 | EUBREC_0489 | [ACR74280.1](https://www.ncbi.nlm.nih.gov/entrez/viewer.fcgi?db=protein&val=ACR74280.1) | WP_041253833.1 | GH36 | bifunctional α-galactosidase/sucrose kinase | cytoplasmic | 444806 - 452160 | raffinose catabolism |
| gtfA | EUBREC_0490 | [ACR74281.1](https://www.ncbi.nlm.nih.gov/entrez/viewer.fcgi?db=protein&val=ACR74281.1) | WP_041253835.1 | GH13 | sucrose phosphorylase | cytoplasmic |  |  |
| EUBREC_RS02255 | EUBREC_0499 | [ACR74290.1](https://www.ncbi.nlm.nih.gov/entrez/viewer.fcgi?db=protein&val=ACR74290.1) | WP_012741407.1 | GH31 | α-glucosidase | cytoplasmic | 457874 - 465787 | α-glucan processing |
| EUBREC_RS02260 | EUBREC_0500 | [ACR74291.1](https://www.ncbi.nlm.nih.gov/entrez/viewer.fcgi?db=protein&val=ACR74291.1) | WP_012741408.1 | GH31 | α-glucosidase | cytoplasmic |  |  |
| EUBREC_RS02435 | EUBREC_0546 | [ACR74336.1](https://www.ncbi.nlm.nih.gov/entrez/viewer.fcgi?db=protein&val=ACR74336.1) | WP_012741453.1 | GH13 | α-amylase | cytoplasmic | 499769 - 508246 | α-glucan processing |
| EUBREC_RS02725 | EUBREC_0616 | [ACR74405.1](https://www.ncbi.nlm.nih.gov/entrez/viewer.fcgi?db=protein&val=ACR74405.1) | WP_012741522.1 | GH05 | endo β-glucanase | extracellular | 559765 - 564334 | unknown |
| EUBREC_RS03960 | EUBREC_0891 | [ACR74661.1](https://www.ncbi.nlm.nih.gov/entrez/viewer.fcgi?db=protein&val=ACR74661.1) | WP_012741763.1 | GH13 | α-glucosidase | cytoplasmic | 795844 - 839894 | unknown |
| EUBREC_RS03965 | EUBREC_0892 | [ACR74662.1](https://www.ncbi.nlm.nih.gov/entrez/viewer.fcgi?db=protein&val=ACR74662.1) | WP_012741764.1 | GH13 | α-glucosidase | cytoplasmic |  |  |
| EUBREC_RS04585 | EUBREC_1006 | [ACR74768.1](https://www.ncbi.nlm.nih.gov/entrez/viewer.fcgi?db=protein&val=ACR74768.1) | WP_012741869.1 | GH53 | endo-β-1,4-galactanase | cytoplasmic | 894538 - 905070 | unknown |
| EUBREC_RS04525 | EUBREC_1014 | [ACR74776.1](https://www.ncbi.nlm.nih.gov/entrez/viewer.fcgi?db=protein&val=ACR74776.1) | WP_012741877.1 | GH13 | cyclomaltodextrinase | cytoplasmic | 901678 - 918352 | unknown |
| EUBREC_RS04640 | EUBREC_1040 | [ACR74802.1](https://www.ncbi.nlm.nih.gov/entrez/viewer.fcgi?db=protein&val=ACR74802.1) | WP_012741903.1 | GH08 | reducing end exo-xylosidase | cytoplasmic | 932672 - 940992 | (arabino)xylan catabolism |
| EUBREC_RS04650 | EUBREC_1042 | [ACR74804.1](https://www.ncbi.nlm.nih.gov/entrez/viewer.fcgi?db=protein&val=ACR74804.1) | WP_012741905.1 | GH43 | arabinofuranosidase or  β-xylosidase | cytoplasmic |  |  |
| EUBREC_RS04660 | EUBREC_1044 | [ACR74806.1](https://www.ncbi.nlm.nih.gov/entrez/viewer.fcgi?db=protein&val=ACR74806.1) | WP_012741907.1 | GH03 | β-glucosidase | cytoplasmic | 941998 - 969395 | unknown |
| EUBREC_RS04785 | EUBREC_1072 | [ACR74834.1](https://www.ncbi.nlm.nih.gov/entrez/viewer.fcgi?db=protein&val=ACR74834.1) | WP_012741935.1 | GH94 | Cellobiose phosphorylase | membrane | – | unknown |
| EUBREC_RS05800 | EUBREC_1308 | [ACR75068.1](https://www.ncbi.nlm.nih.gov/entrez/viewer.fcgi?db=protein&val=ACR75068.1) | WP_012742167.1 | GH25 | *N*-acetylmuramidase | unknown | – | peptidoglycan processing |
| EUBREC_RS05835 | EUBREC_1317 | [ACR75077.1](https://www.ncbi.nlm.nih.gov/entrez/viewer.fcgi?db=protein&val=ACR75077.1) | WP_012742176.1 | GH42 | β-galactosidase | cytoplasmic | 1191924 - 1194782 | unknown |
| EUBREC_RS06135 | EUBREC_1385 | [ACR75145.1](https://www.ncbi.nlm.nih.gov/entrez/viewer.fcgi?db=protein&val=ACR75145.1) | WP_012742244.1 | GH02 | β-galactosidase or α-L-arabinopyranosidase | cytoplasmic | 1253174 - 1267004 | (arabino)galactan catabolism |
| EUBREC_RS06140 | EUBREC_1386 | [ACR75146.1](https://www.ncbi.nlm.nih.gov/entrez/viewer.fcgi?db=protein&val=ACR75146.1) | WP_012742245.1 | GH53 | endo-1,4-β-galactanase | cytoplasmic |  |  |
| EUBREC_RS06480 | EUBREC_1456 | [ACR75210.1](https://www.ncbi.nlm.nih.gov/entrez/viewer.fcgi?db=protein&val=ACR75210.1) | WP_012742309.1 | GH23 | soluble lytic murein transglycosylase precursor | unknown | – | peptidoglycan processing |
| MalQ | EUBREC_1840 | [ACR75584.1](https://www.ncbi.nlm.nih.gov/entrez/viewer.fcgi?db=protein&val=ACR75584.1) | WP_041254060.1 | GH77 | 4-α-glucanotransferase | cytoplasmic | 1709187 - 1724557 | unknown |
| EUBREC_RS09110 | EUBREC_2020 | [ACR75761.1](https://www.ncbi.nlm.nih.gov/entrez/viewer.fcgi?db=protein&val=ACR75761.1) | WP_012742857.1 | GH02 | β-galactosidase | cytoplasmic | 1903453 - 19103 | unknown |
| EUBREC_RS09175 | EUBREC_2034 | [ACR75775.1](https://www.ncbi.nlm.nih.gov/entrez/viewer.fcgi?db=protein&val=ACR75775.1) | WP_012742871.1 | GH04 | β-galactosidase | cytoplasmic | 1920068 - 1927141 | unknown |
| EUBREC_RS16640 | EUBREC_2079 | [ACR75820.1](https://www.ncbi.nlm.nih.gov/entrez/viewer.fcgi?db=protein&val=ACR75820.1) | WP_012742916.1 | GH25 | endo-N-acetylmuramidases (muramidases) | unknown | – | peptidoglycan processing |
| EUBREC_RS10425 | EUBREC_2308 | [ACR76042.1](https://www.ncbi.nlm.nih.gov/entrez/viewer.fcgi?db=protein&val=ACR76042.1) | WP_012743137.1 | GH73 | mannosyl-glycoprotein endo-β-N-acetylglucosaminidase | extracellular | 2114231 - 2174853 | unknown |
| EUBREC_RS11065 | EUBREC_2453 | [ACR76184.1](https://www.ncbi.nlm.nih.gov/entrez/viewer.fcgi?db=protein&val=ACR76184.1) | WP_012743278.1 | GH42 | β-galactosidase | cytoplasmic | – | unknown |
| EUBREC_RS11070 | EUBREC_2454 | [ACR76185.1](https://www.ncbi.nlm.nih.gov/entrez/viewer.fcgi?db=protein&val=ACR76185.1) | WP_118375268.1 | GH51 | α-L-arabinofuranosidase | cytoplasmic | – | unknown |
| EUBREC_RS11615 | EUBREC_2578 | [ACR76309.1](https://www.ncbi.nlm.nih.gov/entrez/viewer.fcgi?db=protein&val=ACR76309.1) | WP_012743398.1 | GH43 | β--xylosidase | cytoplasmic | 2446431 - 2455601 | unknown |
| EUBREC_RS11670 | EUBREC_2592 | [ACR76323.1](https://www.ncbi.nlm.nih.gov/entrez/viewer.fcgi?db=protein&val=ACR76323.1) | WP_012743412.1 | GH31 | sulfoquinovosidase | cytoplasmic | 2456329 - 2475367 | plant sulfolipid catabolism |
| EUBREC_RS11870 | EUBREC_2636 | [ACR76367.1](https://www.ncbi.nlm.nih.gov/entrez/viewer.fcgi?db=protein&val=ACR76367.1) | WP_012743453.1 | GH25 | *N*-acetylmuramidase | extracellular | – | peptidoglycan processing |
| EUBREC_RS16695 | EUBREC_2755 | [ACR76485.1](https://www.ncbi.nlm.nih.gov/entrez/viewer.fcgi?db=protein&val=ACR76485.1) | WP_012743513.1 | GH24 | probable phage-related lysozyme | unknown | – | peptidoglycan processing |
| EUBREC_RS12640 | EUBREC_2816 | [ACR76545.1](https://www.ncbi.nlm.nih.gov/entrez/viewer.fcgi?db=protein&val=ACR76545.1) | WP_012743573.1 | GH94 | cellobiose phosphorylase | cytoplasmic | 2691565 - 2701108 | β-glucan catabolism |
| EUBREC_RS12645 | EUBREC_2817 | [ACR76546.1](https://www.ncbi.nlm.nih.gov/entrez/viewer.fcgi?db=protein&val=ACR76546.1) | WP_041254661.1 | GH94 | cellobiose phosphorylase | membrane |  |  |
| EUBREC_RS12660 | EUBREC_2820 | [ACR76549.1](https://www.ncbi.nlm.nih.gov/entrez/viewer.fcgi?db=protein&val=ACR76549.1) | WP_012743577.1 | GH03 | β-glucosidase | cytoplasmic |  |  |
| EUBREC_RS12940 | EUBREC_2879 | [ACR76608.1](https://www.ncbi.nlm.nih.gov/entrez/viewer.fcgi?db=protein&val=ACR76608.1) | WP_012743635.1 | GH05 | β-glucosidase | cytoplasmic | – | glucoside catabolism |
| EUBREC_RS14800 | EUBREC_3299 | [ACR77025.1](https://www.ncbi.nlm.nih.gov/entrez/viewer.fcgi?db=protein&val=ACR77025.1) | WP_012744052.1 | GH25 | *N*-acetylmuramidase | unknown | 3124805 - 3139240 | cell wall remodelling |
| EUBREC_RS15170 | EUBREC_3387 | [ACR77113.1](https://www.ncbi.nlm.nih.gov/entrez/viewer.fcgi?db=protein&val=ACR77113.1) | WP_012744129.1 | GH36 | α-galactosidase | cytoplasmic | – | unknown |
| EUBREC_RS16365 | EUBREC_3678 | [ACR77404.1](https://www.ncbi.nlm.nih.gov/entrez/viewer.fcgi?db=protein&val=ACR77404.1) | WP_012744387.1 | GH13 | α-glucosidase | cytoplasmic | 3419975 - 3424813 | α-glucan processing |
| EUBREC_RS16400 | EUBREC_3687 | [ACR77413.1](https://www.ncbi.nlm.nih.gov/entrez/viewer.fcgi?db=protein&val=ACR77413.1) | WP_012744396.1 | GH01 | 6-phospho-β-glucosidase | cytoplasmic | 3428107 - 3441165 | unknown |
